# Targeting of Mammalian Glycans Enhances Phage Predation in the Gastrointestinal Tract

**DOI:** 10.1128/mBio.03474-20

**Published:** 2021-02-09

**Authors:** Sabrina I. Green, Carmen Gu Liu, Xue Yu, Shelley Gibson, Wilhem Salmen, Anubama Rajan, Hannah E. Carter, Justin R. Clark, Xuezheng Song, Robert F. Ramig, Barbara W. Trautner, Heidi B. Kaplan, Anthony W. Maresso

**Affiliations:** a Department of Molecular Virology and Microbiology, Baylor College of Medicine, Houston, Texas, USA; b Department of Human Genetics, Baylor College of Medicine, Houston, Texas, USA; c Department of Biochemistry, Emory Comprehensive Glycomics Core, Emory University School of Medicine, Atlanta, Georgia, USA; d Michael E. Debakey Veterans Affairs Medical Center, Houston, Texas, USA; e Department of Medicine, Baylor College of Medicine, Houston, Texas, USA; f Department of Microbiology and Molecular Genetics, McGovern Medical School, University of Texas Health Science Center at Houston, Houston, Texas, USA; University of Texas Southwestern Medical Center Dallas

**Keywords:** *E. coli*, bacteriophage therapy, bacteriophages, multidrug resistance, pathobiont, pathogens

## Abstract

The human gastrointestinal mucosal surface consists of a eukaryotic epithelium, a prokaryotic microbiota, and a carbohydrate-rich interface that separates them. In the gastrointestinal tract, the interaction of bacteriophages (phages) and their prokaryotic hosts influences the health of the mammalian host, especially colonization with invasive pathobionts. Antibiotics may be used, but they also kill protective commensals. Here, we report a novel phage whose lytic cycle is enhanced in intestinal environments. The tail fiber gene, whose protein product binds human heparan sulfated proteoglycans and localizes the phage to the epithelial cell surface, positions it near its bacterial host, a type of locational targeting mechanism. This finding offers the prospect of developing mucosal targeting phage to selectively remove invasive pathobiont species from mucosal surfaces.

## Abstract (precis)

Invasive pathobionts or microbes capable of causing disease can reside deep within the mucosal epithelium of our gastrointestinal tract. Targeted effective antibacterial therapies are needed to combat these disease-causing organisms, many of which may be multidrug resistant.

## INTRODUCTION

The human mucosal surface is a diverse community composed of bacteria, viruses, fungi, and human epithelial, immune, and stem cells. The chemistry of the surface is complex, commonly composed of proteins, lipids, nucleic acid, and small molecule metabolites (1, 2). The pH, ionic environment, and physio-mechano properties also contribute to this dynamic surface. All of these factors influence the biology and, in turn, disease and treatment. Research over the last decade has revealed the importance of mucosal homeostasis (3–5). Invasive pathobionts may colonize, grow, and cause acute or chronic infections, some of which may become systemic and life threatening (6, 7). The bacterial composition of this intestinal community varies in association with human illnesses such as cancer, diabetes, neurological illness, obesity, and cardiovascular disease (8–12).

The meteoric rise of multidrug resistance threatens a return to preantibiotic days by 2050 (13). Drug-resistant bacteria such as the pandemic extraintestinal pathogenic Escherichia coli (ExPEC) sequence type 131 (ST131) are associated with systemic infections of the urinary tract, brain, peritoneum, peripheral organs, blood, and indwelling devices, resulting in 9 million infections per year (14–16). These strains readily colonize the human intestine, which can become a reservoir, prior to extraintestinal infection (17).

The rise of multidrug resistance has precipitated the search for alternative antibacterial approaches, including the use of bacteriophages (phages), which are bacterial viruses whose infection cycle leads to lysis of bacteria and thus have been used to treat infections. Although phage therapy has been used in Eastern Europe for decades, only in the past few years has it been employed for compassionate use cases in the United States and Europe and in clinical trials (18–20). In theory, phage offer some advantages over antibiotics, one of which is that they can be specific toward a given species of bacteria, potentially sparing the microbiome (21). Also, they are “generally regarded as safe” (GRAS), allowing them to be given in high doses with low to no associated toxicity (22). Phage are also diverse, as phage mixtures can be used to broaden the range and/or reduce the frequency of resistance (23). However, their greatest attribute has been speculated to be their abundance, estimated to be 10^31^ on Planet Earth. The phage genosphere—the collection of all genes and their unique biology—is sometimes referred to as “viral dark matter” and may encode novel features that allow for enhanced lytic activity toward bacterial pathogens (24–26). Reasoning that this genosphere may include phage with unique phenotypes that facilitate lytic activity against pathobionts in mucosal environments, we report here a novel podovirus that binds human heparan sulfated proteoglycans and is effective at predation of its E. coli host in complex gastrointestinal (GI) microenvironments.

## RESULTS

### The gastrointestinal tract is prohibitive to phage therapy.

**FIG 1:**
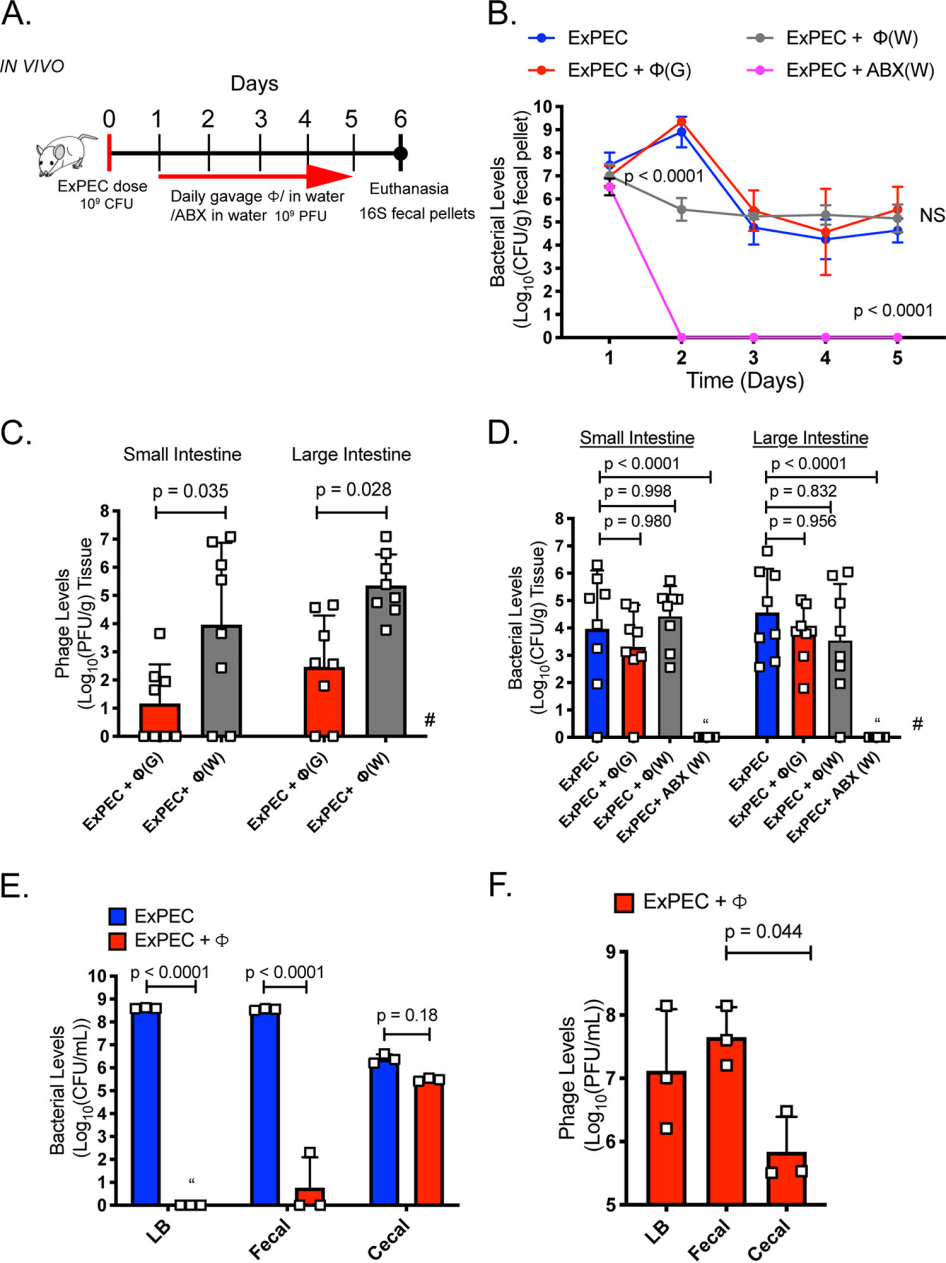
The gastrointestinal tract is prohibitive to phage therapy. (A) Mice were orally gavaged on day 0 with 10^9^ CFU of ExPEC JJ1901 and then monitored for colonization by plating fecal pellets for colony counts on selective medium (LB plus chloramphenicol). Starting on day 1, groups received either daily phage dose (10^9^ PFU), phage in water (10^9^ PFU/ml), or antibiotic (ABX), 2 mg/ml ampicillin in water, until day 5. On day 6, mice were euthanized and their organs were homogenized and plated for CFU levels or PFU levels (plaque assay). (B) Intestinal (fecal) colonization of ExPEC. Colored dots represent means. ExPEC plus phage gavage, ExPEC+Φ(G); ExPEC plus phage in water, ExPEC+Φ(W); ExPEC plus antibiotic in water, ExPEC+ABX(W). (C) Intestinal tissue and contents phage levels. (D) Intestinal tissue and contents ExPEC levels. *N* = 8 to 10. Open squares represent individual mice, bars represent means. #, missing values due to histological analysis. (E) ExPEC levels after 4.5 h of growth (shaking, 37°C) in LB, fecal medium, or cecal medium (prepared as described for Fig. S1E in the supplemental material) with or without phage (MOI 10). (F) PFU counts of phage HP3 after 4.5 h of growth in different medium. Open squares represent independent cultures (*N* = 3). Bars represent means ± standard deviation (SD). NS, not significant; “, none detected. One-way ANOVA used for statistical analysis.

Previous studies reported that phage HP3, a lytic myovirus isolated from environmental reservoirs of ExPEC, reduces ST131 bacteremia and disease severity in murine models of infection (27, 28). Since the human gastrointestinal tract is the primary reservoir of ExPEC ST131, we wondered if phage HP3 could act prophylactically to reduce or eliminate ExPEC burden in the intestine. To test this, mice were orally gavaged with an ExPEC ST131 clinical isolate JJ1901, previously used in the mouse model of bacteremia study described above (27), and then treated with phage or an antibiotic as illustrated in Fig. 1A. Untreated mice sustained stable bacterial colonization during the course of the experiment (6 days) (Fig. 1B). When phage HP3 was given to animals via water or a daily gavage, the levels of ExPEC were indistinguishable from that of the untreated control at the end of the experiment. An uptick in CFU was noted on day 2; however, all groups leveled out by day 3 until the end of the experiment. No ExPEC was detected at any time point in the antibiotic-treated group. Interestingly, phage HP3 was detected and active as determined via plaque assay by plating the stool of phage-treated mice on an overlay of ExPEC bacteria, even on day 4, indicating that the lack of ExPEC reduction was not due to a lack of delivery to the intestinal environment or to inactivation of the phage (see Fig. S1A in the supplemental material). It should also be noted that despite having higher levels of phage upon gavage during days 1 to 4, the phage was no more effective at removing ExPEC than phage given in the water, suggesting that in this experiment, there was no dose-dependent effect of phage. In addition, as many as 10^5^ PFU/g phage were found in the murine intestinal tissue (including cecum and colon) on day 6, the final day of the study, yet there was little to no clearing of ExPEC compared to that in these tissues of the untreated control (Fig. 1C and D). Importantly, antibiotic treatment significantly reduced the number of operational taxonomic units (OTUs) and diversity, as determined by the Shannon diversity index, which takes into account species richness and distribution (Fig. S1B and C) (*P* = 0.007, *P* = 0.009). Also, a principal-component analysis (PCA) of beta diversity demonstrated that mice in the antibiotic cohort clustered together and away from the untreated and phage groups, suggesting antibiotics had a more profound effect on the microbiome than phage (Fig. S1D). Finally, we assessed phage killing in a modified “cecal medium” (CM) that is derived from the cecal contents from recently euthanized mice. These cecal contents were pooled and homogenized in sterile saline solution and then centrifuged to remove large particulates. This medium is designed to simulate the luminal complexity of the mammalian intestine, since it contains fecal matter, a microbiome, mucus, and likely many of the small molecules and proteins present in an intestinal lumen (Fig. S1E). Whereas phage HP3 completely abolished ExPEC in LB (nearly a 9-log drop in levels and no detectable live bacteria) and nearly abolished it in a slurry of fecal pellets taken from the same murine host (∼8-log drop in levels), there was little to no phage-based killing in CM, despite recovering nearly 10^6^ to 10^7.5^ PFU/ml of phage (Fig. 1E and F). These results mirrored those from the ExPEC colonization model and indicate there is a factor(s) present in the mammalian GI that inhibits this lytic phage.

10.1128/mBio.03474-20.1 FIG S1 (A) Intestinal (fecal) phage colonization. (B) OTU from 16S rRNA gene analysis of fecal pellets on day 6. (C) Shannon diversity index values shown from 16S rRNA gene analysis of fecal pellets on day 6. (D) Beta diversity shown using unweighted UniFrac of principal-coordinate analysis (PCoA) plot of 16S rRNA gene from fecal pellets day 6. ExPEC+Φ(G), ExPEC plus phage gavage; ExPEC+Φ(W), ExPEC plus phage in water; ExPEC+ABX(W), ExPEC plus antibiotic in water. Open squares represent individual mice in panels A to C, and closed circles represent individual mice in panel D. *N* = 10. Bars indicate means ± SD. One-way ANOVA used for significance. (E) *Ex vivo* cecal model. Cecal contents were removed from just-euthanized mice, pooled, and homogenized in sterile 0.09% saline solution (1:5, mg/ml dilution). Homogenate was centrifuged to remove large particulates (2,000 × *g* for 30 s). The supernatant fluid was used for phage killing assays with ExPEC JJ1901 (∼10^6^ CFU), and phage HP3 (10^7^ PFU) added at the same time (MOI, 10) and then incubated at 37°C with shaking (255 rpm) for 4.5 h. After incubation, ExPEC CFU was determined by selectively plating for the bacteria on LB plus chloramphenicol plates. Download FIG S1, DOCX file, 0.3 MB. Copyright © 2021 Green et al. 2021 Green et al. This content is distributed under the terms of the Creative Commons Attribution 4.0 International license.

### The inhibitory component is mucin.

**FIG 2:**
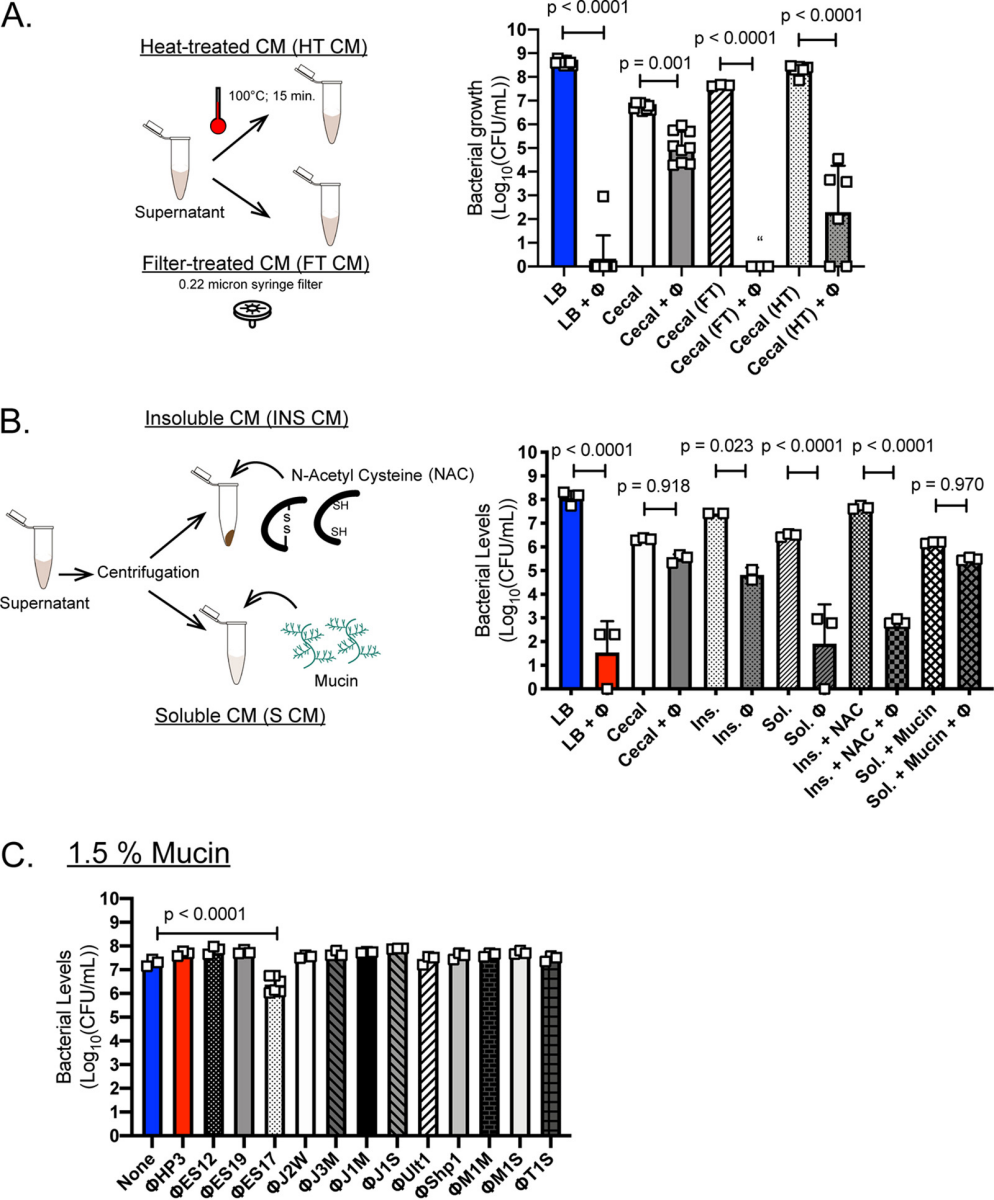
The inhibitory component is mucin. (A) Cecal medium (CM) was prepared as described for Fig. S1E, heat treated (HT CM) at 100°F for 20 min or centrifuged (6,000 × *g*; 5 min), filtered (0.22-μm filter treated or FT), and then used for a 4.5-h growth assay as described for Fig. S1E. (B) Cecal medium was centrifuged at a high speed (9,000 × *g*, 5 min) separated into pellet (insoluble [Ins]) and supernatant (soluble [Sol]). The Ins was resuspended in saline ± NAC (*N*-acetyl cysteine, 5 mg/ml), the soluble ± mucin (porcine gastric mucin, 1% [wt/vol]). ExPEC levels are after a 4.5-h assay. (C) ExPEC levels following a 4.5-h assay screen with phages from Table 1 in mucin (1.5% [wt/vol] in LB). For all experiments, MOI of 10. Open squares represent independent cultures. Mean (bars) ±SD shown throughout, *N* = 3 to 9. “, none detected. One-way ANOVA used for statistical analysis.

We wished to understand the reasons phage HP3 was ineffective in this intestinal microenvironment. We next tested whether the inhibition might be related to the presence of live bacterial microbiota in CM. However, ExPEC killing with phage was not enhanced with removal of the microbiota with a broad range of antibiotics, including inhibitors of protein synthesis, cell wall, and DNA synthesis (see Fig. S2A and B). (Note that the antibiotics efficiently killed a commensal, antibiotic-sensitive, E. coli that was spiked into the CM (Fig. S2C)). Arriving at no resolution as to what the inhibitory factor may be, we decided to test more drastic treatments for their ability to restore phage killing in CM. First, the CM was heat treated (HT CM) (Fig. 2A). Interestingly, heat treatment led to a >6-log improvement in ExPEC killing by phage HP3 (*P* < 0.0001). Similarly, when CM was filter treated (0.22-μm filter) (FT CM), there was no detectable level of ExPEC in the medium after treatment with phage HP3 (Fig. 2A), and this observation was extended to another phage showing inhibition in CM, EC1 (Fig. S2D) (*P* < 0.0001). Thus, the inhibitory component was large (retained on 0.22-μm filter) and sensitive to boiling.

10.1128/mBio.03474-20.2 FIG S2 ExPEC levels after 4.5 h. growth in LB or cecal medium with or without phage (MOI, 10) incubated with or without antibiotic (ABX), chloramphenicol (Cm; 10 μg/ml) (A) or with ampicillin sodium salt (AMP; 100 μg/ml) or ciprofloxacin (CIP; 10 μg/ml) (B). (C) Commensal E. coli ECN (antibiotic sensitive) levels after cecal assay described above. (*N* = 3 to 5). (D) ExPEC levels after cecal medium (CM) was prepared as described for Fig S1E, centrifuged (6,000 × *g*, 5 min), filtered (0.22-μm filter treated [FT]), and used for a 4.5-h growth assay as described for Fig. S1E. (*N* = 3 to 6). (E) Mice were gavaged daily with *N*-acetyl cysteine for 2 weeks. ExPEC was gavaged on day 0 according to the protocol for Fig. 1A and then treated with daily gavage of phage HP3 starting on day 1 to 5. On day 6, mice were euthanized, and organs were homogenized and plated for phage counts and ExPEC counts. (F) Small intestine (tissue) and contents ExPEC levels. (G) Large intestine (tissue) and contents ExPEC levels. (*N* = 9). (H) ExPEC levels in LB plus different mucin concentrations (0%, 0.5%, 1%, and 1.5% [wt/vol]) with or without phage HP3 (MOI, 10) from 0 to 8 h growth. (I) ExPEC levels following a 4.5-h assay screen with phages from Table 1 in LB. (J) ExPEC levels after 4.5 h of growth with phages HP3 or ES17 (MOI of 10) in LB plus different concentrations of mucin (0%, 0.25%, 0.5%, 0.75%, 1%, 1.25%, 1.5%, 1.75%, and 2%) (*N* = 3). NS, not significant; “, none detected. Open squares represent independent cultures or mice. Bars indicate means ±SD. One-way ANOVA used for significance. Download FIG S2, DOCX file, 0.5 MB. Copyright © 2021 Green et al. 2021 Green et al. This content is distributed under the terms of the Creative Commons Attribution 4.0 International license.

We reasoned intestinal mucins might fit this profile due to their highly associative and sticky properties (captured on a filter), and as proteins, they would be sensitive to heat. Mucins are glycoproteins found throughout the gastrointestinal system which form a layer between the intestinal epithelial cells (IECs) and the commensal or pathogenic microbiota (29). Also, they can function as receptors for microbes (30). To test whether mucins were inhibiting bacterial killing by phage, we devised another method whereby CM was separated via high-speed centrifugation into soluble (S CM) and insoluble (INS CM) forms (Fig. 2B). We hypothesized that INS CM would contain mucin, since large intestinal mucins are normally present in this portion, and be inhibitory to phage killing, whereas the S CM would not have these properties (31). Indeed, phage killing in unprocessed CM or INS CM was inhibited to a greater extent than in S CM (Fig. 2B). To more directly test the hypothesis that mucin was the inhibitory factor, the mucolytic drug *N*-acetyl cysteine (NAC) was added to INS CM, and porcine gastric mucin (1.5% [wt/vol]) was added to S CM. Indeed, a 5-log reduction in phage killing of ExPEC was observed in INS CM upon addition of NAC, which also improved killing to that seen in S CM (*P* < 0.0001). Perhaps more compelling, the addition of mucin to S CM abrogated bacterial killing by phage to levels originally observed in CM alone (not significant, *P* = 0.970). These results indicate that the inhibitor of phage killing in cecal medium is intestinal mucin.

Along these lines, E. coli is known to use mucins as a source of carbon (32). We reasoned that a murine host colonized with ExPEC may see a bloom upon NAC treatment due to the drug liberating the mucins for bacterial consumption and thus serve as a system to test if the reduction in aggregated mucin would promote phage HP3’s ability to kill ExPEC. Upon treatment with NAC for 2 weeks, ExPEC levels in the small intestine of mice were increased, and phage HP3 reduced ExPEC levels, although it was not significant (Fig. S2E and F). A similar trend was observed in the large intestine (Fig. S2G). The less pronounced effect in the large intestine compared to that in the small intestine may be due to the thickness of mucus and thus the lower likelihood for NAC to be effective at breaking up this mucus. Also, NAC is known to be rapidly absorbed in the small intestinal tissue, thereby losing its effect in the more distal large intestine (33, 34).

### Discovery of a mucin-enhanced phage.

Reasoning that human sewage or the feces of animals may contain phage that have evolved to target their host in high-mucin environments, such as the intestinal tract, we screened our phage library (35) and other phages (Table 1) recently isolated from these environments for enhanced activity in LB containing 1.5% mucin (Fig. 2C). This is the same concentration that prevented phage HP3 activity in soluble cecal medium and was shown to provide strong inhibition in LB for up to 8 h (Fig. S2H). Surprisingly, only a single phage, designated phage ES17, significantly reduced bacteria in the LB mucin medium (Fig. 2C) (*P* < 0.0001, approximately 1 log). Phage ES17 was active in mucin despite being much less effective (>3 log) than phages HP3, J2W, Ult1, Shp1, or M1S, which completely killed ExPEC to undetectable levels in LB medium alone (Fig. S2I). Consistent with these data, when the amount of mucin was varied from 0% to 2% and phages HP3 and ES17 were compared for lytic activity against ExPEC, phage HP3 was highly effective as the concentration of mucin was lowered to <0.5% but completely inhibited at higher levels (Fig. S2J). However, phage ES17 was most effective at concentrations in which HP3 was inactive (0.5% to 1% mucin) and least active at concentrations lower or higher than in this range. Taken together, these data suggest that phage ES17 harbors unique properties that facilitate its ability to efficiently be lytic in the presence of mucin.

**TABLE 1:** Properties of E. coli phages used in mucin screen

Property	Phage name:											
	ES12	ES17	ES19	J2W	J3M	J1M	J1S	Ult1	Shp1	M1M	M1S	T1S
Source	Human	Human	Human	Human	Human	Human	Human	Human	Sheep	Rabbit	Rabbit	Pigs
Source location	Sewage	Sewage	Sewage	Sewage	Sewage	Sewage	Sewage	Sewage	Animal facility	Animal facility	Animal facility	Animal facility
Isolation strain	JJ2050	JJ2547	DS104	JJ2528	JJ2528	JJ2528	JJ2528	JJ1901	JJ1901	JJ1901	JJ1901	JJ1901
Plaque size (mm)	0.5	0.5–1.0	0.5	0.5	0.5	0.5	0.5	0.5–1.0	0.5	0.5	0.5	0.5
Plaque morphology	Clear	Clear, halo	Clear, halo	Clear, halo	Clear	Clear	Clear	Clear	Clear	Clear	Clear	Clear
ST131 lysed (no. [%])^*a*^	9/13 (69)	9/13 (69)	11/13 (85)					12/13 (92)	12/13 (92)	11/13 (85)	12/13 (92)	10/13 (77)
Accession no.	MN508614	MN508615	MN508616									

a ExPEC ST131 library of isolates described previously (35).

### Phage ES17 is a C3-type phage whose activity is enhanced by mucin.

**FIG 3:**
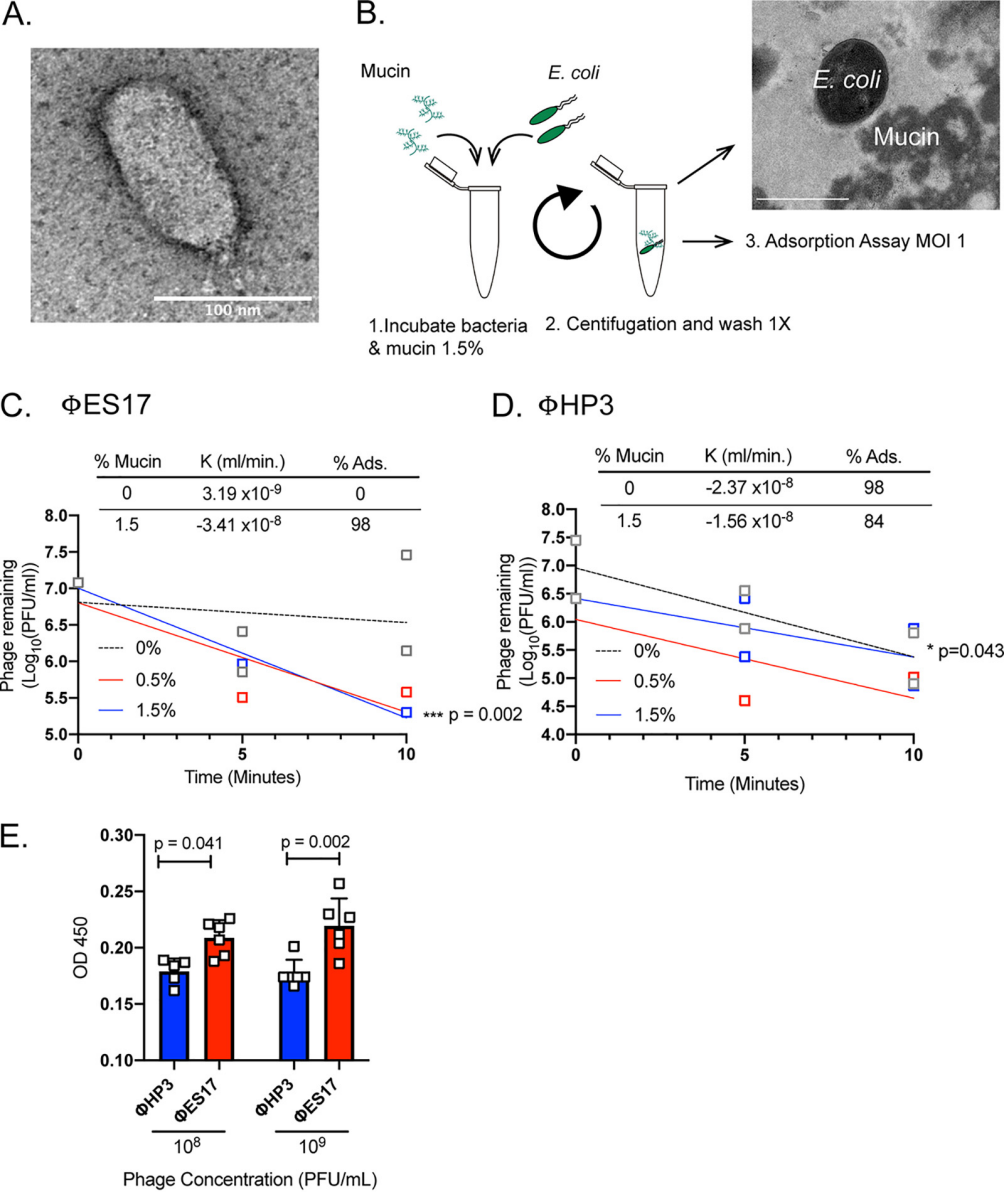
Phage ES17 is a C3-type phage with avidity for mucin. (A) Transmission electron microscopy (TEM) image of phage ES17. (B) Mucin (0%, 0.5% and 1.5% [wt/vol]) was added to the ExPEC cultures for 10 min at RT with shaking (255 rpm). The cultures were centrifuged (6,000 × *g* for 5 min) and gently washed with PBS. Adsorption curve (MOI, 1) was constructed using mid-log-phase cultures and samples taken every 5 min for 10 min. ExPEC coated with mucin pictured in TEM image. White bar, 1 μm. (C) Adsorption phage ES17. (D) Adsorption phage HP3. Adsorption constant (*K*) and percent adsorbed (%Ads) in 10 min for 0% and 1.5% mucin indicated above the curves. Regression lines generated from mean values of phage remaining for time points indicated. Open squares represent individual values from separate cultures. Dashed lines, 0% mucin; red lines, 0.5% mucin; blue lines, and 1.5% mucin in bacteria. Two biological replicates for 0% and 1.5%. (E) ELISA with 1.5% mucin-coated wells. Different phages were incubated overnight on coated wells and then washed, and the assay was performed as described in Materials and Methods. Open squares represent biological replicates. Different concentrations of phages incubated on wells indicated on *x* axis. *N* = 6. Bars represent means ±SD shown throughout. Simple linear regression was used to calculate if slope is significantly nonzero for panels C and D. Two-way ANOVA used for statistical analysis in panel E.

We sought to understand the molecular mechanism of phage ES17’s enhanced ability to find and lyse its bacterial host in mucin. Phage ES17 was determined to be a double-stranded (dsDNA) virus of the order *Caudovirales*, family *Podoviridae*, genus *Kuravirus* (see the supplemental methods in Text S2). PhiEco32, another Kuravirus phage, shows close genetic similarity to ES17 (see Fig. S3A) (36). Kuravirus phages have elongated C3-type capsids, an uncommon morphology, short tail fibers, and small genomes (37, 38). ES17 has these similar morphological characteristics (capsid of >100 nm) (Fig. 3A). ES17 has a small genome size consisting of 75,007 bp with 123 predicted open reading frames (ORFs) (MN508615) (Fig. S3B).

10.1128/mBio.03474-20.3 FIG S3 (A) Graphical representation of BLAST genomic comparison analysis of phage ES17 (bottom axis; MN508615) and phage phiECo32 (top axis; EU330206.1) using Kablammo web-based software (90). Trapezoids drawn between the axis indicate individual BLAST alignments between the two sequences. The stronger alignments are shaded darker. (B) ES17 genome organization. Download FIG S3, DOCX file, 0.2 MB. Copyright © 2021 Green et al. 2021 Green et al. This content is distributed under the terms of the Creative Commons Attribution 4.0 International license.

To determine why this phage is distinct from other phages that lack activity in mucin-rich environments, we examined the ability of phages ES17 and HP3 to adsorb to their E. coli hosts. Previous data had shown that 98% of HP3 was adsorbed in 10 min, whereas only 32% of ES17 was adsorbed in that time (35). Additionally, no major differences were previously found between ES17 and HP3 as determined by one-step growth curve parameters such as burst size (ES17, 36; HP3, 60) or latent period (ES17, 32 min; HP3, 22.5 min) (27, 35). We wondered whether the addition of mucin could improve phage ES17 adsorption and inhibit the adsorption of phage HP3. A modified adsorption assay was utilized for this experiment using ExPEC and mucin. Briefly, the bacteria were incubated in different concentrations of mucin (0% to 1.5%), pelleted, and washed to remove any mucin that did not adhere to the bacterial surface (Fig. 3B). Next, a standard adsorption assay was conducted with phages ES17 and HP3. Interestingly, phage ES17 showed no adsorption to ExPEC in 10 min in the absence of mucin; however, if first incubated with 1.5% mucin, adsorption increased to 98% (Fig. 3C). In contrast, 98% of phage HP3 was adsorbed without mucin, and the adsorption dropped to 84% in its presence (Fig. 3D). Finally, we adapted an enzyme-linked immunosorbent assay (ELISA)-like approach to determine if phage ES17 preferred binding to surfaces coated with mucin, a hypothesis consistent with our data. Indeed, when mucin was bound to an ELISA plate, phage ES17 bound to the mucin surface at higher levels than phage HP3 (Fig. 3E) (*P* = 0.041 and *P* = 0.002). Taken together, using these different approaches, the data suggest that phage ES17 binds mucin, a property that may enhance its ability to infect E. coli in mucin-rich environments.

### Phage ES17 binds human heparan sulfated proteoglycans.

Phage ES17 harbors an enhanced ability relative to that of other E. coli phages to find its bacterial host in environments in which carbohydrates are a prominent chemical component (examples from above include cecal medium and mucin-rich broth). ES17’s putative tail fiber protein (ES17-TFP) showed high similarity, based on a BLAST analysis (64% similar; E value, 0), to a tail fiber protein in another lytic podophage, the T7-like bacteriophage LM33_P1 (YP_009324518.1), which also targets ST131 strains (39) (see Fig. S4A). T7-like phage tail fibers have been shown to possess endosialidases that target surface sugars, such as capsule-forming polysaccharides (40). A BLAST analysis revealed that ES17-TFP contains a putative pectinesterase (E value, 7.45e−03; 369 bp). This domain was only found in four other phages, myPSH1131, myPSH2311, vB_EcoS_Golestand, and LM33_P1, and of those, only myPSH1131 has it in the same tail fiber protein as ES17 (Fig. S4B).

10.1128/mBio.03474-20.4 FIG S4 (A) BLAST comparison analysis of ES17-TFP (top axis) and phage LM33_P1 tail fiber (bottom axis, YP_009324518.1). Trapezoids drawn between the axis indicate individual BLAST alignments between the two sequences. The stronger alignments are shaded darker. (B) ES17 TFP (tail fiber protein; 3,066 bp) pectinesterase domain blast analysis of showing similarity of domain to other phages. (C) Structures show the predicted structure of TFP tail fiber protein (blue), the structure of K5 lyase (PDB 2X3H; red). Identical residues between K5 lyase and ES17 tail fiber protein are yellow. Magnified sugar-binding domains shown in boxes. (D) Structures of heparosan or K5 capsule and heparan sulfate derivative. (E) Protein SDS-PAGE gel showing purified ES-TFP pooled elutions and benchmark protein ladder (lane 1). Pooled elutions from 2-liter purification of TFP (lane 2). Approximate size of TFP, 107 kDa. (F) Glycan microarray analyses of 138 glycans from purified porcine gastric mucin in relative fluorescent units (RFU) using purified ES17-TFP as the glycan binding protein (GBP) (ii), 560 defined glycans from the Consortium for Functional Glycomics using TFP as GBP (ii), and 170 charged glycosaminoglycans (GAG), including oligomers from hyaluronic acid, chondroitin sulfates and heparan sulfate using TFP as GBP (iii). (G) Immunofluorescent staining of ES17 and HP3 phages fixed on a slide using antibodies generated against HP3 (red). 40× magnification. (H). HIEMs uninfected (i) or infected with EAEC 042 (ii)or pretreated with phage ES17 prior to EAEC (iii). Image magnification, ×60. (iv) EAEC attached to HIEMs per field view. Means ± SD shown. Squares indicate individual biological replicates from independent cultures. Figure created with BioRender software. Download FIG S4, DOCX file, 0.5 MB. Copyright © 2021 Green et al. 2021 Green et al. This content is distributed under the terms of the Creative Commons Attribution 4.0 International license.

A structural analysis of modeled ES17-TFP showed a high similarity to a phage K5 lyase binding domain (E value = 4e−12). The predicted structure of ES17-TFP (blue) and K5 lyase (red) are pictured in Fig. S4C (PDB 2X3H) with identical residues colored yellow. Phage K5 binds K5 capsular polysaccharide and acts as a K5 polysaccharide lyase (41). The K5 E. coli capsule is made of a repeating disaccharide that is identical to the precursor of heparin and heparan sulfate (HS), a linear polysaccharide present in glycosaminoglycans (heparan sulfate proteoglycans [HSPGs]) (Fig. S4D). These proteoglycans are found on mammalian cells and in mucus (42). Also, mucins with similar structures to that of heparan sulfate/heparin (α-linked GlcNAc or *N*-acetyl-d-glucosamine) are present intestinally and found in porcine gastric mucin (PGM) (43).

We reasoned that ES17’s enhanced activity might be due to an ability to bind mammalian polysaccharides found on glycoproteins, as other groups have found with different phage types (44–48). However, none of these groups had identified heparan sulfate proteoglycans, ubiquitous glycoproteins present at the basement membranes and surfaces of various cell types, as likely receptors for this interaction (49, 50). Using this mechanism, phage could localize to its host, thereby explaining its enhanced activity in a mucin-rich environment. We wished to extend these observations further and specifically pinpoint the exact type of carbohydrate that might mediate the hypothesized activity. To test this idea, we cloned and purified ES17-TFP (Fig. S4E) and assessed the ability of purified ES17-TFP to bind to a glycan array containing more than ∼860 unique glycan structures from porcine gastric mucin (PGM), glycosaminoglycans (GAGs) and a variety of synthetic and naturally sourced glycans generated by the Consortium for Functional Glycomics (CFG) (Text S1). No or very low relative fluorescent units (RFU), a proxy for binding, was observed for a wide array of mammalian glycans, including those purified from porcine gastric mucin (Fig. S4Fi to ii). However, surprisingly, there was an increase of several orders of magnitude in RFU (RFU > 2,000) observed for binding to the GAGs containing heparan sulfate (identification numbers [ID no.] 64 to 173) but not the structurally similar GAGs hyaluronic acid no. 1 to 20 or chondroitin sulfate no. 21 to 63 (Fig. S4Fiii). The finding that purified ES17-TFP binds human heparan sulfated proteoglycans provides a possible mechanism to explain why phage ES17 demonstrates enhanced activity in intestinal environments.

10.1128/mBio.03474-20.6 TEXT S1 Supplemental methods. Download Text S1, DOCX file, 0.05 MB. Copyright © 2021 Green et al. 2021 Green et al. This content is distributed under the terms of the Creative Commons Attribution 4.0 International license.

### ES17 binds to the surface of human intestinal enteroids.

**FIG 4:**
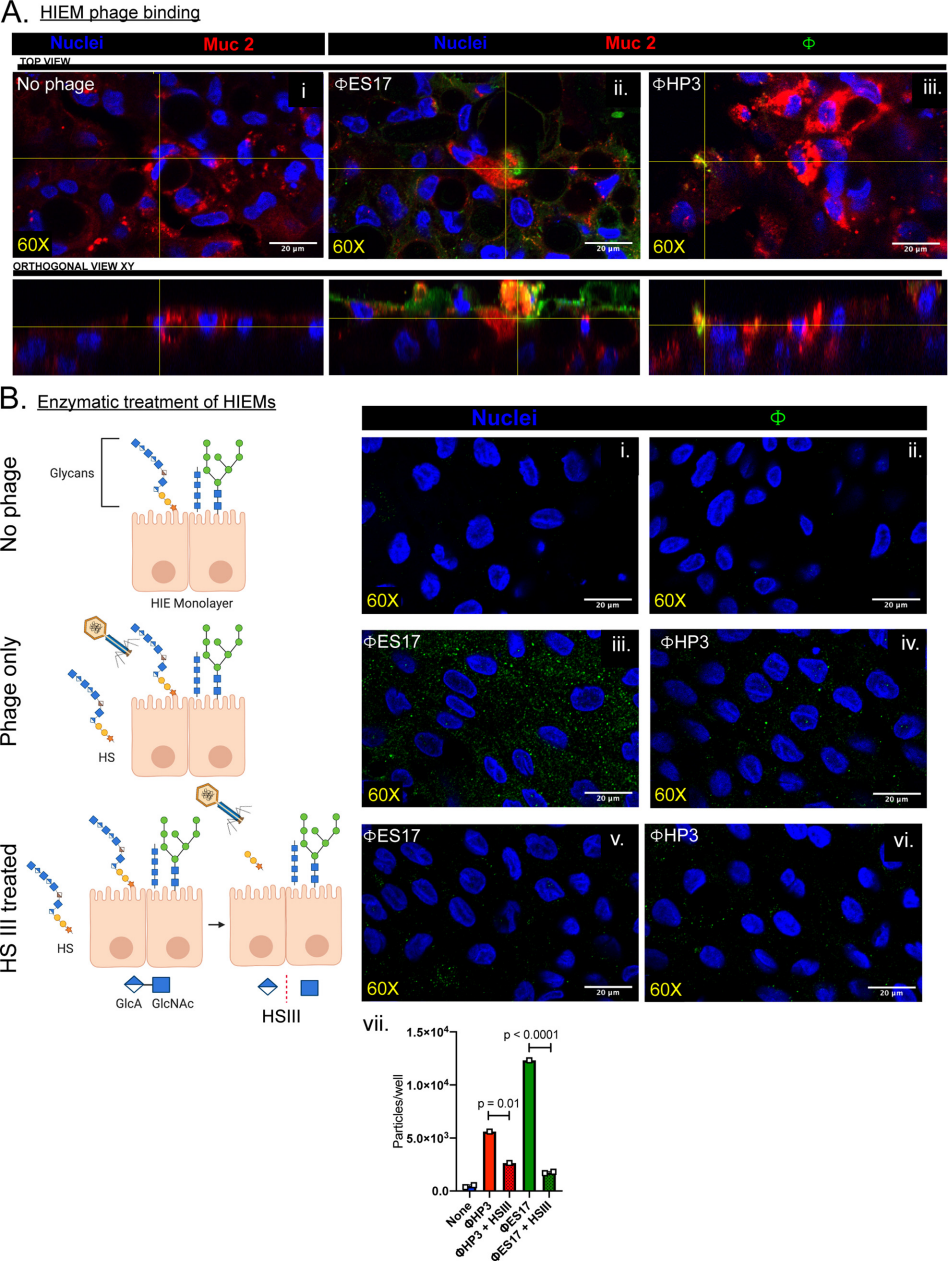
ES17 binds to the surface of human intestinal enteroids (HIEMs). Differentiated HIEMs were incubated with phages (10^8^ PFU/ml for 1 h) at 37°C, 5% CO_2_, in a humidified incubator. Afterwards, HIEMs were washed, fixed in Clark’s solution, stained, and imaged for phage (Alexa Fluor 488; green), intestinal cells (DAPI; blue), and Muc2 (Alexa Fluor 594; red). Image magnification, ×60. Orthogonal view XY shown below. (A) No phage added (i), phage ES17 added (ii), and phage HP3 added (iii). (B) To selectively removed HSPG from GAG chains, enteroid cultures were pretreated with heparinase III (Hep III; 2 U/ml) for 2 h at 37°C, 5% CO_2_, in a humidified incubator and then incubated with or without phage as described above. (B) No phage (i and ii), phage ES17 added (iii), phage HP3 added (iv), Hep III-treated HIEMs incubated with ES17 (v), and Hep III-treated HIEMs incubated with HP3 (vi). Image magnification, ×60. (vii) Quantification of particles (phage) per well using FIJI software. Means ±SDs shown (*N* = 1 to 2). Open squares represent independent cultures. One-way ANOVA used for statistical analysis panel B created with BioRender software.

Human intestinal enteroids (HIEs) are organotypic higher-order cultures that have become popular as surrogates to model the human intestine. They can be grown as 3-dimensional structures complete with a lumen and crypt/villus axis or as 2-dimensional monolayers that facilitate host-pathogen interactions (51, 52). These cultures are also useful because they express a variety of glycans found in the human intestine, including mucins and proteoglycans (51). Human intestinal enteroid monolayers (HIEMs) were derived from colonic stem cells following differentiation for 5 days in high-Wnt medium. Phage ES17 or HP3 was added to confluent HIEMs for 1 h, extensively washed, and visualized by immunofluorescence microscopy using antibodies raised against each phage. Little to no detectable phage HP3 was observed on the HIEMs intestinal epithelial cell (IEC) surface, though antibodies generated robust signal and specificity toward the phage when HP3 was fixed on slides alone (Fig. 4A and S4G). Phage ES17 (green) bound evenly to the IECs on the apical side, including areas where prominent Muc2 (red) localization was observed, but also on areas where there was no Muc2 staining (Fig. 4Aii). To determine whether phage ES17 bound to the IECs of HIEMs via heparan sulfate, we pretreated the HIEMs with heparinase III to enzymatically remove HSPGs and then assessed phage binding. Indeed, HIEMs treated with heparinase significantly reduced the levels of bound ES17, both qualitatively and quantitatively (Fig. 4Bi to vii) (*P* < 0.0001). This finding is consistent with the purified tail fiber protein specifically binding to HSPGs. The localization of ES17 to both the mucus layer and to the IEC surface was by binding to HSPGs, which likely position the phage to be in the exact location needed to find its bacterial target in the intestinal microenvironment. To show that ES17 when bound to HIEMs could still infect bacteria, we utilized the diarrhea-causing pathogen enteroaggregative Escherichia coli (EAEC), which adheres to HIEMs robustly in an aggregative mesh-like pattern unlike ExPEC (53). For this experiment, we precoated HIEMs with phage ES17, washed them, as detailed above, and then infected them with EAEC strain 042. HIEMs were fixed and stained using a Giemsa-Wright stain to visualize cells and bacteria, as previously described (53, 54). Infected HIEMs showed robust bacterial adhesion to cells with an aggregative phenotype (Fig. S4Hii, red arrows). Phage-coated HIEMs, however, showed significantly reduced EAEC on the surface of the organoid (Fig. S4Hiii and iv) (*P* = 0.007), demonstrating that bound ES17 is still infectious to this significant biofilm-forming pathogen.

### Phage ES17 kills ExPEC in the mammalian intestine.

**FIG 5:**
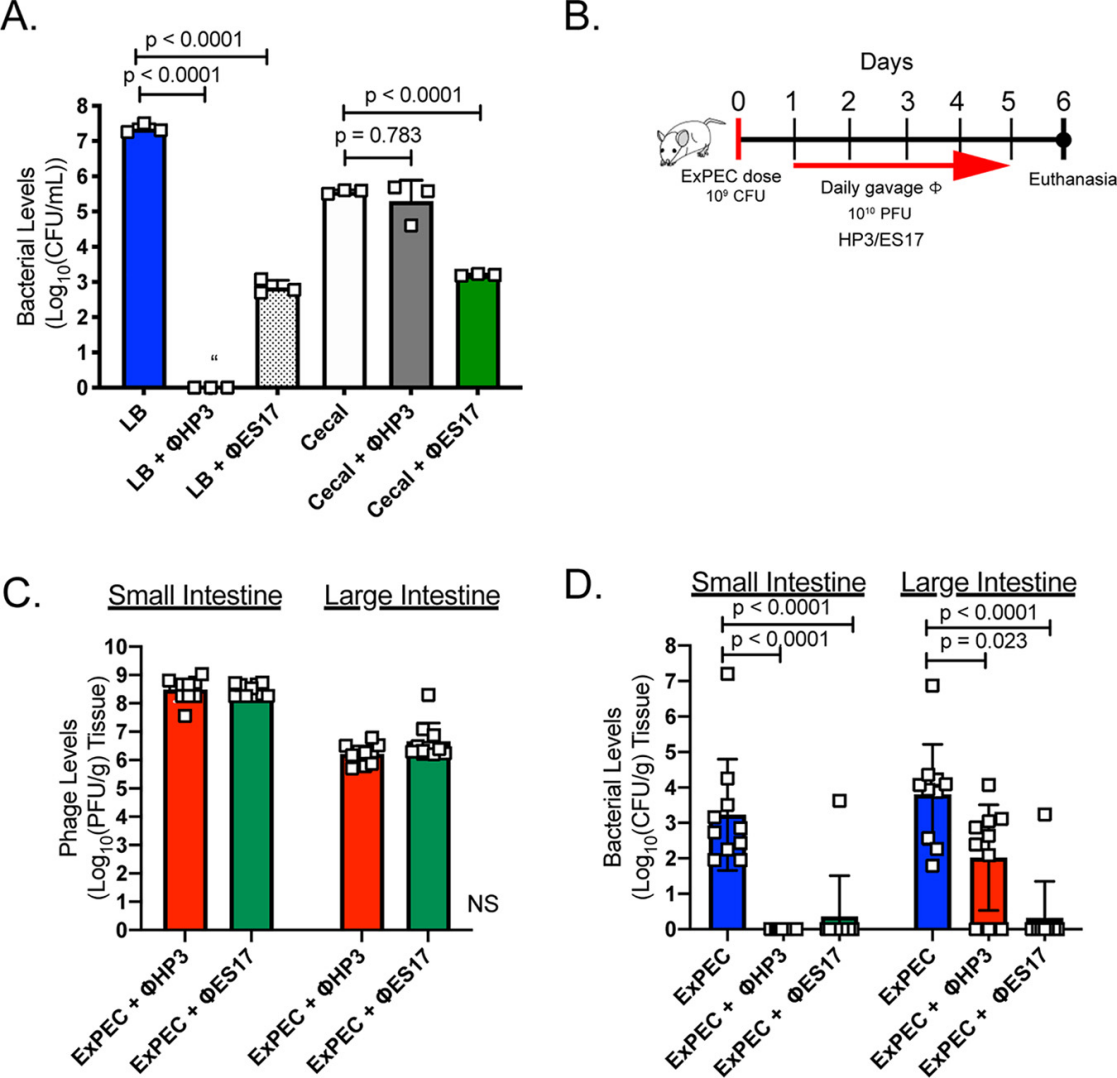
Phage ES17 kills in the mammalian intestine. (A) ExPEC levels after 4.5-h growth assay as described for Fig. S1E in cecal medium or LB (MOI, 10). Bars indicate the means, and open squares represent independent cultures. *N* = 3. (B) Mice were orally gavaged on day 0 with 10^9^ CFU of ExPEC JJ1901. Starting on day 1, mice received either daily dose (gavage) of phages (HP3 or ES17 at 10^10^ PFU) or none until day 5. On day 6, mice were euthanized and their organs were homogenized and plated for CFU levels or plaque assay for PFU levels. (C) Intestinal (tissue) and contents phage levels. (D) Intestinal (tissue) and contents ExPEC levels. *N* = 10 mice per group. Bars indicate the means ± SD, and open squares indicate individual mice. “, none detected. One-way ANOVA used for statistical analysis.

The finding that phage ES17 demonstrated enhanced lytic activity in the presence of mucins, was the best of several screened phages in a mock luminal environment rich in mucins, and binds the human organotypic culture IECs via HSPGs prompted an examination into whether this phage could overcome the intestine-induced inhibition of phage lytic activity toward colonized ExPEC that was observed for phage HP3. We first tested if ES17 was effective in cecal medium. Indeed, phage ES17 showed a 2.5-log improvement in ExPEC removal in this environment compared to that of phage HP3 (Fig. 5A) (*P* < 0.0001). There was no effect on the number of OTUs or the Shannon diversity index in this experiment, suggesting phage ES17 was highly selective at removing only the target ExPEC strain (see Fig. S5A and B). We next tested the effect of phage ES17 on ExPEC in a murine intestine. Animals were colonized with ExPEC as in Fig. 1A and treated with either phage ES17 or HP3 (Fig. 5B). The dose of phage was also increased from 10^9^ PFU to 10^10^ PFU to also evaluate if giving more phage would improve HP3’s ability to reduce ExPEC, especially in more proximal segments, as the phage slowly moves through the alimentary canal. Examination of the small and large intestines on day 6 showed phage levels were high across all groups (10^6^ to 10^8^ PFU/g intestinal tissue) (Fig. 5C). Animals treated with phage HP3 had no detectable CFU in small intestinal tissue (Fig. 5D) but had indistinguishable levels from those of the untreated controls in the cecum. This reduction and improved phage levels in the gut may be due to a log increase in daily phage dose given to animals (10^10^ PFU) compared to that used for Fig. 1 (10^9^ PFU). In contrast, every animal treated with phage ES17, except one, had no detectable levels of ExPEC in either the small or large intestine, suggesting that this lytic phage possesses a unique ability to target ExPEC in complex mucosal environments such as the large intestine.

10.1128/mBio.03474-20.5 FIG S5 (A) OTU from 16S rRNA gene analysis of cecal medium after incubation with phage ES17. (B) Shannon diversity index values shown of cecal medium after incubation with phage ES17. *N* = 3. Means ± SD are shown. Squares represent independent cultures. One-way ANOVA used for significance. NS, not significant. Download FIG S5, DOCX file, 0.2 MB. Copyright © 2021 Green et al. 2021 Green et al. This content is distributed under the terms of the Creative Commons Attribution 4.0 International license.

## DISCUSSION

A limitation of all antibiotics is their broad killing activity and no inherent features to function well in complex human environments, including human blood, urine, or at mucosal surfaces throughout the body. In particular, the gastrointestinal tract (GIT) is a highly complex system made up of diverse organs and tissues and, within that, a diversity of cell types. Some of the cells and factors found at the mucosal surface may influence intestinal capacity, including intestinal epithelial cells, enteroendocrine cells, stem cells, mucus-secreting goblet cells, antimicrobial peptides (AMPs; from Paneth cells), and secretory immunoglobulin A (55–59). To add to this complexity, the GIT is colonized by a large number of microbes, including archaea, fungi, protists, and bacteria that have coevolved with the host, termed the human gut microbiota (55, 60). A bacteriophage would have to navigate through this complex ecosystem to find and infect its host. Since there are estimated to be >10^10^ PFU/g of phages already present in the GIT, this suggests that phages can thrive in this environment (61, 62). Here, we report a novel antibacterial targeting strategy that seems to have evolved to position a lytic bacteriophage in the exact niche as its bacterial host. Our results indicate that (i) phage HP3 that is lytic toward E. coli ST131 *in vitro* and is very effective at eliminating bacteremia in a murine model of sepsis is ineffective when tested for this same property in the murine intestinal tract, (ii) the inhibition is due to intestinal mucins, (iii) a medium designed to mimic the luminal environment can be used to identify phage with enhanced lytic activity in such an environment, and (iv) a podovirus phage, isolated from human wastewater, overcomes this inhibitory activity due to an enhanced ability of the phage to bind to heparan sulfated proteoglycans present in mucus or immobilized on the surface of intestinal epithelial cells, which likely drives the positional targeting of the phage to the exact ecological niche as the host bacterium. In addition, our data reveal that this treatment, compared to antibiotic treatment, did not alter the intestinal diversity of the microbiota. Taken together, these data suggest a new mechanism of predation by a phage, one which may be highly useful for the killing of bacteria in intestinal environments.

A potential narrow or limited ecological range of phages has not been explored as much as their narrow host range capabilities. Using an experimental process of elimination, we determined that mucin can greatly inhibit phage infection. Mucins are composed of tandem repeats of serine and threonine that act as attachment sites for *o*-linked glycans (*N*-acetlygalactosamine, *N*-acetylglucosamine, fucose, galactose, and sialic acid) (30, 63). Gel-forming mucins, which make up the mucus intestinal layer are large polymers (up to 40 MDa) of mucins attached via disulfide linkages (30). When we incubated phages with porcine gastric mucin, composed of the gel-forming mucin MUC 5AC (64), we saw reduced or no bacterial killing. However, when we added *N*-acetyl cysteine (NAC), which is known to exert a mucolytic effect by reducing the disulfide bonds that keep these mucin polymers together, bacterial killing was restored (33, 65). NAC is a drug has been tested and used in the clinical setting to treat syndromes, including cystic fibrosis (CF) and chronic obstructive pulmonary disease (COPD). In both of these cases, it loosens thick mucus in lungs, but it can also serve as a treatment for acetaminophen overdose due to its antioxidant action (65–67). Perhaps this drug could also be used as an adjuvant to phages to help treat patients with bacterial infections in mucin-rich ecosystems such as the gut or the lungs of CF patients. We tested oral NAC treatment in mice and found it to be more effective in improving phage-mediated bacterial killing in the small intestine than in the large intestine. NAC has been shown to be rapidly absorbed following oral dosing after only 60 min (34). An oral dose of NAC has a short half-life of 2.5 h and 10% bioavailability in the gut (34). It is likely that NAC did not exert a mucolytic effect on the distal large intestine. Interestingly, we observed elevated ExPEC levels in intestinal tissue with NAC treatment. It is possible that some pathogens like ExPEC may thrive in environments with a reduced mucus layer.

Another possibility, distinct from adjuvating phage with NAC, would be to search for phages that have evolved phenotypes that enhance their ability to encounter their bacterial host in a specific ecosystem, such as the GIT. Phage ES17, originally isolated from human sewage, is a member of the family *Podoviridae* and has a C3-like elongated capsid morphology with paddle-like short tail fibers (68). Phage ES17 showed a significant killing effect in cecal medium (CM) and in a mouse model of intestinal colonization compared to that for phage HP3. Phage ES17 bound mucin significantly better, and mixing bacteria with mucin increased phage adsorption. There is precedence for this concept. The bacteriophage-adhering-to-mucus (BAM) model proposes that phages bind to mucus via *hoc* proteins or Ig (immunoglobulin fold-like) domains present on the capsid proteins of phages similar to T4 (44, 45, 69). This mechanism was suggested to explain how phages bind to the metazoan mucosal surface and increase the probability of encountering a bacterial host, likely via a unique type of controlled diffusion that increases spurious interactions with its bacterial target (44, 45). These studies showed that T4 adsorption increased in the presence of mucin from 65% to 80% absorbed phage within 10 min (45). The adsorption changes in phage ES17 in the presence of mucin were all or nothing, from 0% to 98% within 10 min. This suggests that mucin may be a type of “bridge receptor” for phage ES17, since this phage is ineffective at killing in the absence of mucin, even at very high levels of phage. Such a model would make sense in the context of E. coli embedded in a mucin matrix, perhaps breaking down mucin as a source of carbon and thus binding it, thereby allowing the phage to adapt a capsular polysaccharide binding mechanism to structurally related sugars that are prominent on proteoglycans.

**FIG 6:**
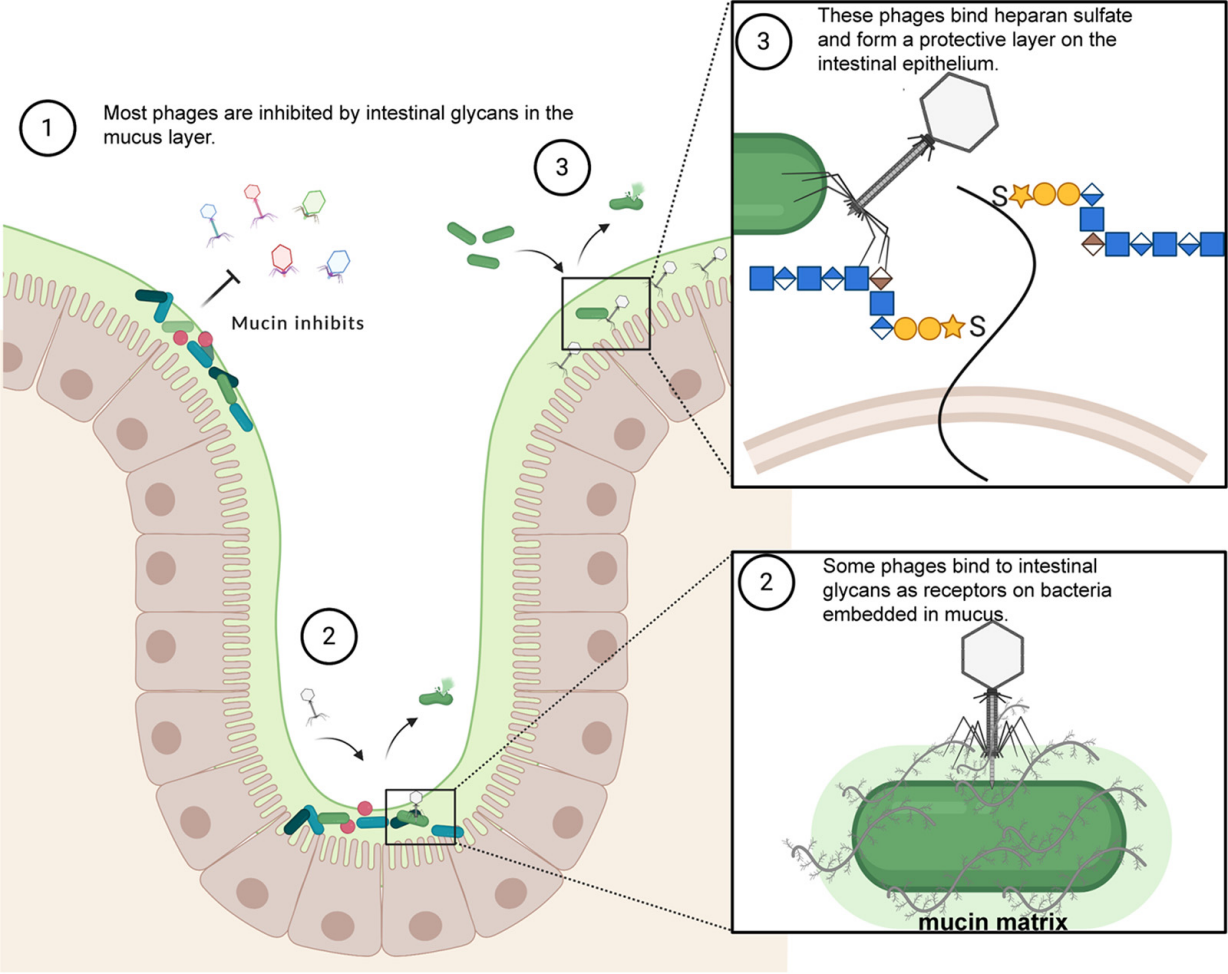
Model showing (1) mucins from the intestinal mucus layer inhibit phage infection, (2) phage ES17 can bind to mucin and utilize other intestinal glycans as a receptor to infect and kill mucus-embedded bacteria, and (3) phages like ES17 can be utilized to coat the intestinal epithelium by binding heparan sulfate glycans to protect from invasive pathogen infection. Adapted from *“Keystone Gut Microbiota Species Provide Colonization Resistance to Invading Bacteria”*, by BioRender.com (2020). Retrieved from https://app.biorender.com/biorender-templates.

Phages have been shown to have a variety of carbohydrate-binding proteins present on tail fibers in order to bind different sugars present on the bacterial cell surface (70). Some of the most diverse are capsular depolymerases, which bind and break down capsular carbohydrates secreted by bacteria (71). These capsular depolymerases are diverse because of the diversity of bacterial capsule types. Capsule types have been shown to mimic some components of the intestinal system, including sugars present in the mucus layer (72, 73). Bacteria can use these mechanisms to subvert the immune system and to allow for the colonization of hosts (72, 74). Phage ES17 possessed a protein with a putative capsular depolymerase domain in a tail fiber protein. However, we could only detect an interaction with a human intestinal sugar, the glycosaminoglycan sugar heparan sulfate. This suggests a model whereby phage ES17 adapted to “colonize” the mucosal environment by binding to mammalian sugars. Heparan sulfate proteoglycans (HSPGs) are expressed on the surfaces of many types of cells and prominently on the surfaces of intestinal epithelial cells (75). One of the more tantalizing discoveries here was that human enteroids derived from colonic stem cells specifically bound phage ES17 but not another E. coli phage, HP3. HSPGs consist of repeating units of sulfated polysaccharides—heparan sulfate (HS). HS is a linear polysaccharide that begins as the precursor heparan (disaccharide of α1,4-linked *N*-acetylglucosamine [GlcNAc] and β1,4-linked glucuronic acid [GlcUA]) (76, 77). Subsequent modifications due to sulfation and epimerization lead to the mature form polysaccharide, HS (77). Bacteriophage K5 utilizes a tail spike lyase (*KflA*) to bind and degrade K5 capsule present on E. coli strains (41). Because the K5 capsule (heparoson; β1,4-linked GlcUA and α1,4-linked GlcNAc) polysaccharide has been shown to be structurally identical to heparan sulfate precursor, heparan, this enzyme can also act a heparinase (41, 77–79). K5 heparan lyase cleaves the linkage of *N*-acetylglucosamine and glucuronic acid but is inhibited by sulfated regions (77, 78). Further homology searches showed that the carbohydrate binding domain found in ES17-TFP is structurally homologous to the binding domain present in the tail spike in phage K5. Considering that heparinase III cleaves the same regions where K5 lyase would bind and degrade, ES17-TFP may utilize the same receptor. Indeed, the addition of heparinase III from the soil bacteria Flavobacterium heparinum, which specifically cleaves unmodified (unsulfated) NAC domains (GlcNAc-GlcUA) and NA/NS domains (GlcNS [*N*-sulfo-d-glucosamine] and GlcNAc) of HS, leads to abrogated phage ES17 binding to cells, meaning that ES17 was highly specific for this type of glycan (77, 80). The structural similarity of mucins and heparan sulfate (class III-type α-linked GlcNAc) suggests that this protein may also target these intestinal mucins (43, 81). This interaction is driven by phage ES17’s tail fiber protein, which is not related to the *hoc* proteins or contain an Ig-like fold as predicted from the BAM model. Thus, it would seem that phage ES17 uses a novel mechanism to localize not only to a mucin-rich surface but also directly to the mammalian epithelial surface (Fig. 6). One limitation of this study, however, is that we did not determine how phage ES17 may be able to bind receptors at the cell surface once its tail fiber is bound to mucin or heparan sulfate. We speculate that ES17 can modify these glycans in order to reach these surface receptors and adsorb onto ExPEC.

Nevertheless, this epithelium binding ability allows this phage to reach areas deep in the mucosal surfaces where there may be refuges of bacteria present (82). Several pathogens, including bacteria and human-pathogenic viruses, have been shown to bind to HS and use them as receptors (83–85). Rather remarkably, around the same time as this discovery, our lab has discovered that EAEC also uses HSPGs as the receptor (86). It is tempting to speculate that this property might have been also acquired by phage, as suggested here.

## MATERIALS AND METHODS

### Bacterial strains and phages.

ExPEC ST131 isolate JJ1901 was used in all ExPEC infections, except for those shown in Fig. S1Aiii (JJ2528) in the supplemental material. Both isolates were previously obtained from Jim Johnson (University of Minnesota) (27). Commensal E. coli ECN (Fig. S2C) was isolated from a human fecal sample. Prior to infections, all strains were grown overnight at 37°C from a single colony streaked on an LB agar plate.

Phages HP3, ES12, ES17, ES19, ES21, and ES26 were previously described and characterized (27, 35). Phages 6914, 6915, and 6939 were recently isolated from sewage. All phages described in Table 1 were isolated by single plaque isolation from environmental sources as described previously (27, 35).

### Murine infections.

Mixed ages (6 to 10 months) and sexes of BALB/c mice (Jackson Laboratories, Bar Harbor, ME) were used in mouse models of infection. Mice were kept in a specific-pathogen-free (SPF) environment at Baylor College of Medicine CCM (Center for Comparative Medicine) Taub facility. All methods performed on mice were approved and in accordance with relevant guidelines and regulations from the Guide for the Care and Use of Laboratory Animals (National Institutes of Health) and approved by Baylor College of Medicine’s Institutional Animal Care and Use Committee (protocol AN-6372). For infections, mice were kept in a biohazard facility with sterile food and water. The mice were individually housed during colonization experiments, and bedding was replaced with autoclaved techboard liners for daily fecal collection. For colonization experiments, sample size was determined based on previous colonization experiments in mice (27). Mice received a 10^9^ CFU dose of ExPEC strain JJ1901 via oral gavage. Rodent health was monitored daily for indication of pain or disease. Bacterial colonization (fecal and intestinal) was determined after homogenization, selective plating for the chloramphenicol-resistant strain JJ1901 on LB agar plates containing chloramphenicol, and colony counting. A 6-day time course was established based on previous studies showing consistent colonization of ExPEC after 1 oral dose at 10^9^ CFU (87).

Purified phage in 3% (wt/vol) NaHCO_3_ was administered either via gavage or in water with 5% (wt/vol) sucrose *ad libitum*. All groups received sucrose and NaHCO_3_ in water for consistency. The antibiotic ampicillin (1 g/500 ml) was administered in water. Phage colonization was quantified after dilution of homogenates and serial plating on a double agar overlay assay of the ExPEC strain. Phage verification was determined by observation of plaque morphology compared to that of phage that was inoculated into mouse. Phage present in the native mouse microbiota did not plaque on the ExPEC strain (data not shown).

### *Ex vivo* cecal model.

A modified cecal assay was used for experiments (88). Briefly, cecal contents from just-euthanized mice were pooled and homogenized in sterile 0.09% NaCl solution at a 1:5 dilution (milligrams per milliliter). The homogenate was centrifuged to remove large particulates (2,000 × *g* for 30 s). The supernatant fluid was used for 4.5-h phage killing assays at a multiplicity of infection (MOI) of 10 at 37°C, shaking (255 rpm), as previously described (28). All cecal and mucin experiments were performed using independent bacterial cultures grown up from different colonies streaked on a plate. This was considered a biological replicate. For FS CM, cecal supernatant was centrifuged (6,000 × *g* for 5 min.) and filtered through a 0.22-μm syringe filter. For HT CM, the supernatant was heated at 100°F for 20 min in a hot water bath and then cooled to room temperature (RT) for infections. Insoluble CM and soluble CM (supernatant) were isolated post-high-speed centrifugation (9,000 × *g* for 5 min) of CM. The insoluble pellet was resuspended in sterile 0.09% NaCl solution for infections (IN CM). For the mucin assays, porcine gastric mucin type II (PGM; Sigma-Aldrich) was used at various concentrations diluted in phosphate-buffered saline (PBS). The mucolytic drug *N*-acetyl cysteine (NAC; Sigma-Aldrich, 5 mg/ml) was diluted in PBS for demucolytic assays.

### Mucin-coating adsorption and imaging.

For the adsorption curves, assays were performed at an MOI of 1 using mid-log-phase cultures independently grown from different colonies on a plate (this was considered a biological replicate) as we have done for previous publications to characterize phages ES17 and HP3 (27, 35). Prior to adsorption, PGM (0%, 0.5%, and 1.5% [wt/vol]) was added to the bacterial cultures for 10 min at RT with shaking (255 rpm). The cultures were centrifuged (6,000 × *g* for 5 min) and gently washed with PBS. The adsorption rate constants (*K*s) were determined from the natural log of the slope of the adsorption curve versus the bacterial concentration. Time points were taken every 5 min in order to accurately test simultaneously for the different conditions being assayed.

### Mucin binding ELISA assay.

Clear-walled Immulon 2 HB 96-well microtiter plates (Immunochemistry Technologies, no. 227) were used for the ELISAs. PGM (200 μl of 1 mg/ml) was added to a microtiter plate and incubated at 4°C overnight. The next day, the mucin was removed and wells were washed twice with PBS. The phage was added to wells for 1 h and then washed three times in PBST (PBS with 0.1% Tween 20). The wells were blocked with bovine serum albumin (BSA) and then incubated with antibodies for the phage overnight at 4°C. Following washing steps, a horseradish peroxidase (HRP)-conjugated antibody was added for 1 h. To assess phage binding, 3,3′,5,5′-tetramethylbenzidine (TMB) solution was added until the wells turned light blue, and then a stop solution (2 M H_2_SO_4_) was added. The absorbance was read at 450 nm. Each well was considered a biological replicate for this experiment.

### HIEM infection and imaging.

Human enteroid monolayers (HIEMs) were differentiated for 5 days (>90% confluence) as described previously (54). For experiments, each well containing a HIEM was considered a biological replicate. HIEMs were incubated with phage at 10^8^ PFU/ml in culture differentiation medium for 1 h at 37°C in the presence of 5% CO_2_ in a humidified incubator and then washed in PBS. The HIEMs were fixed in Clark’s solution for 10 min to preserve the mucus layer. The HIEMs were permeabilized and blocked with 5% BSA in 0.1% Triton X-100 in PBS for 30 min at RT. Mucus was detected using antibodies to Muc2 (1:200) (Abcam), and nuclei were stained with 4′,6′-diamidino-2-phenylindole (DAPI) (300 nM) for 5 min at RT. Antibodies against phages HP3 and ES17 were generated from whole-virus (phage) injection into rabbits performed by Pacific Immunology. A 13-week antibody production protocol consisted of 4 immunizations and antiserum collection.

To selectively removed HSPG from GAG chains, enteroid cultures were pretreated with heparinase III (Sigma, 2 U/ml) for 2 h, as described previously (89), followed by the addition of phage. Images were captured using a Zeiss LSM 510 confocal microscope. Represented images were adjusted equally for brightness and contrast using FIJI software version 2.0.0. The images were adjusted equally for brightness and contrast. Particle analysis was used to determine the number of particles per well.

### Statistics.

Statistical analysis was performed using PRISM 8 software. Microbiome 16S data were analyzed using ATIMA (Agile Toolkit for Incisive Microbial Analyses) as described in the supplemental methods in Text S1. For figures with log-transformed data and groups of >2, significance was determined using a one-way analysis of variance (ANOVA) (Fig. 1 to 5) or two-way ANOVA when necessary (Fig. 3E). For multiple-comparison analysis, the secondary test, Tukey, was used. For nontransformed data, a normality test (Shapiro-Wilk) was performed to determine normality before a one-way ANOVA analysis was performed. Significance was determined to be a *P* value of less than or equal to 0.05. Unless otherwise stated, all graphs show the means and standard deviations.
