# Supplementary material for: Targeting of Mammalian Glycans Enhances Phage Predation in the Gastrointestinal Tract
Source: mBio. 2021 Feb 9;12(1):e03474-20. doi: 10.1128/mBio.03474-20 (PMC7885116; doi:10.1128/mBio.03474-20)
Supplement: FIG S1 [file mBio.03474-20-sf001.docx]

**
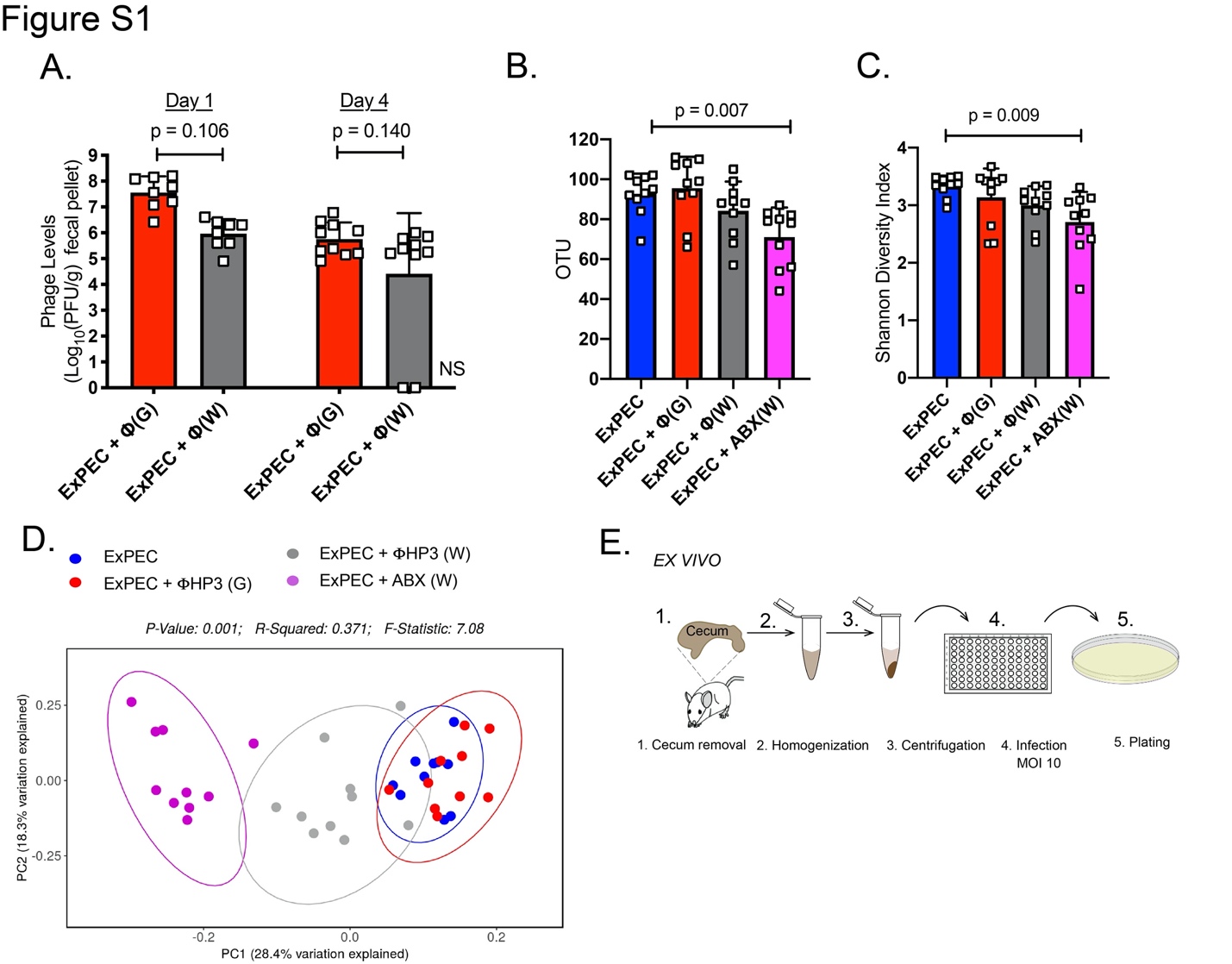
**

**Figure S1** (A) Intestinal (fecal) phage colonization. (B) OTU from 16S rDNA analysis of fecal pellets on day 6. (C) Shannon diversity index values shown from 16S rDNA analysis of fecal pellets on day 6. (D) Beta diversity shown using unweighted Unifrac of principle coordinate analysis (PCoA) plot of 16S rDNA from fecal pellets day 6. Groups: ExPEC Blue; ExPEC + phage gavage (Φ (G)) red; ExPEC + Φ water (W) grey, ExPEC + antibiotic (ABX (W)) purple. Open squares rep. indiv. mice (A-C). Closed circles rep. indiv. mice in Fig S1D. N=10. Mean (bars), ±SD. One-way ANOVA used for significance. (E) *Ex vivo* cecal model. Cecal contents were removed from just euthanized mice, pooled and homogenized in sterile 0.09% saline solution (1:5 mg/ml dilution). Homogenate was centrifuged to remove large particulates (2000G for 30 sec.). The supernatant fluid was used for phage killing assays with ExPEC JJ1901 (~10^6^ CFU) and phage HP3 (10^7^ PFU) added at the same time (MOI 10) and then incubated at 37°C, shaking (255 RPM) for 4.5 hours. After incubation ExPEC CFU was determined by selectively plating for the bacteria on LB + Chloramphenicol plates.
