# Supplementary material for: Targeting of Mammalian Glycans Enhances Phage Predation in the Gastrointestinal Tract
Source: mBio. 2021 Feb 9;12(1):e03474-20. doi: 10.1128/mBio.03474-20 (PMC7885116; doi:10.1128/mBio.03474-20)
Supplement: FIG S2 [file mBio.03474-20-sf002.docx]

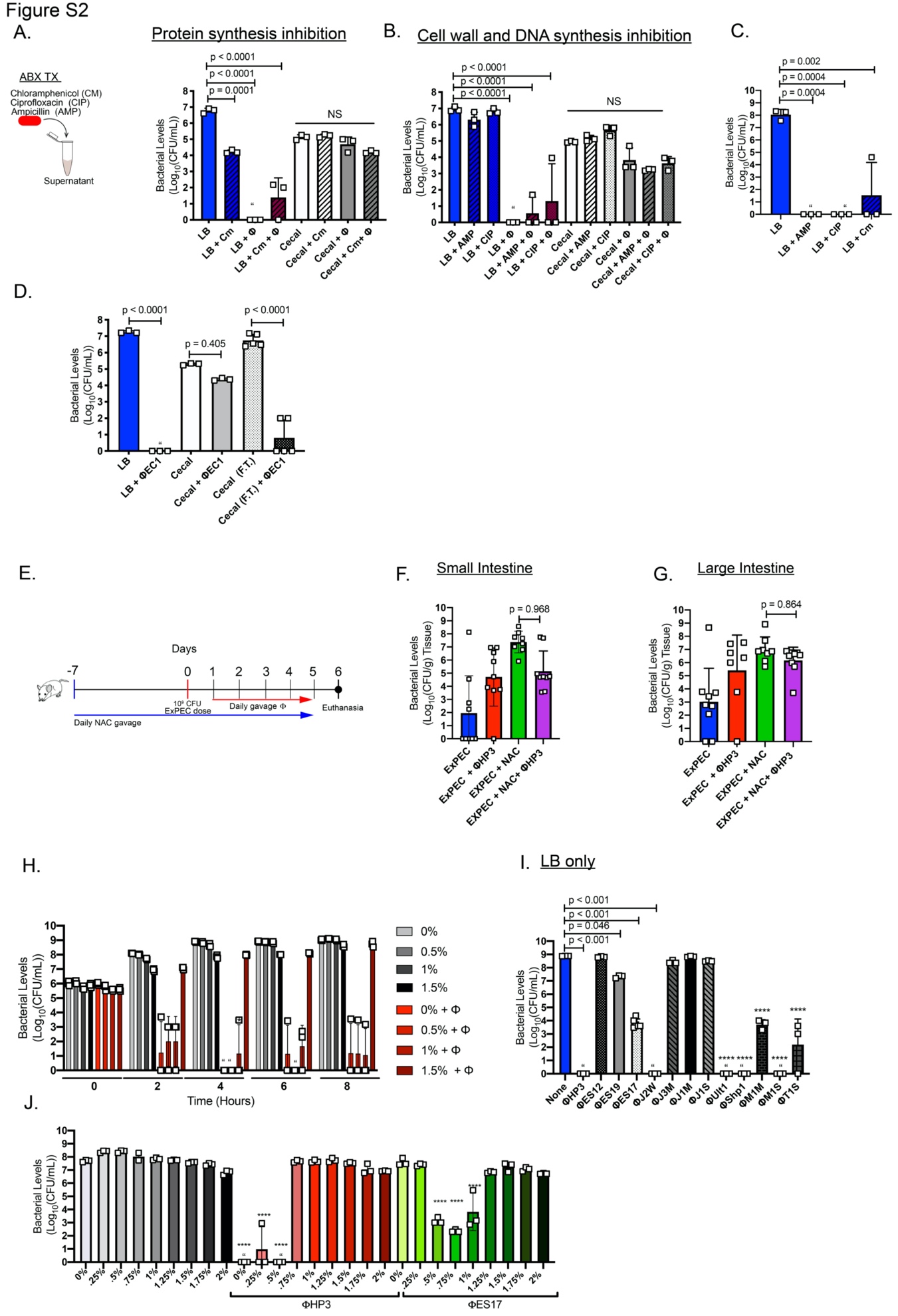


**Figure S2** (A) ExPEC levels after 4.5 hr. growth in LB or cecal medium with or without phage (MOI 10) incubated with or without antibiotic (ABX) chloramphenicol (Cm, 10 ug/mL) or (B) with ampicillin sodium salt (AMP, 100 ug/mL) or ciprofloxacin (CIP, 10 ug/mL). (C) Commensal *E. coli* ECN (antibiotic sensitive) levels after cecal assay described above. (N=3-5). (D) ExPEC levels after Cecal medium (CM) was prepared as described in Figure S1E centrifuged (6000G; 5 min.) then filtered (0.22 micron filter treated or FT) and used for a 4.5 hr. growth assay as described Fig. S1E. (N=3-6). (E) Mice were gavaged daily with N-acetyl cysteine for 2 weeks. ExPEC was gavaged on day 0 following protocol from Figure 1A. Then treated with daily gavage of phage HP3 starting on day 1 to 5. On day 6 mice were euthanized and organs homogenized and plated for phage counts and ExPEC counts. (F) Small intestinal (tissue and contents) ExPEC levels. (G) Large intestinal (tissue and contents) ExPEC levels. (N=9). (H) ExPEC levels in LB + diff. mucin conc. (0%, 0.5%, 1% and 1.5% m/v) with or without phage HP3 (MOI 10) from 0 to 8 hours growth. (I) ExPEC levels following a 4.5 hr. assay screen with phages from Table 1 in LB. (J) ExPEC levels after 4.5 hr. growth with phages HP3 or ES17 (MOI of 10) in LB plus diff. conc. mucin (0%, 0.25%, 0.5%, 0.75%, 1%, 1.25%, 1.5%, 1.75% and 2%) (N=3).

NS not significant.“ = none detected. Open squares rep. indep. cultures or mice. Mean (bars). ±SD. One-way ANOVA used for significance.
