# Supplementary material for: Targeting of Mammalian Glycans Enhances Phage Predation in the Gastrointestinal Tract
Source: mBio. 2021 Feb 9;12(1):e03474-20. doi: 10.1128/mBio.03474-20 (PMC7885116; doi:10.1128/mBio.03474-20)
Supplement: FIG S3 [file mBio.03474-20-sf003.docx]

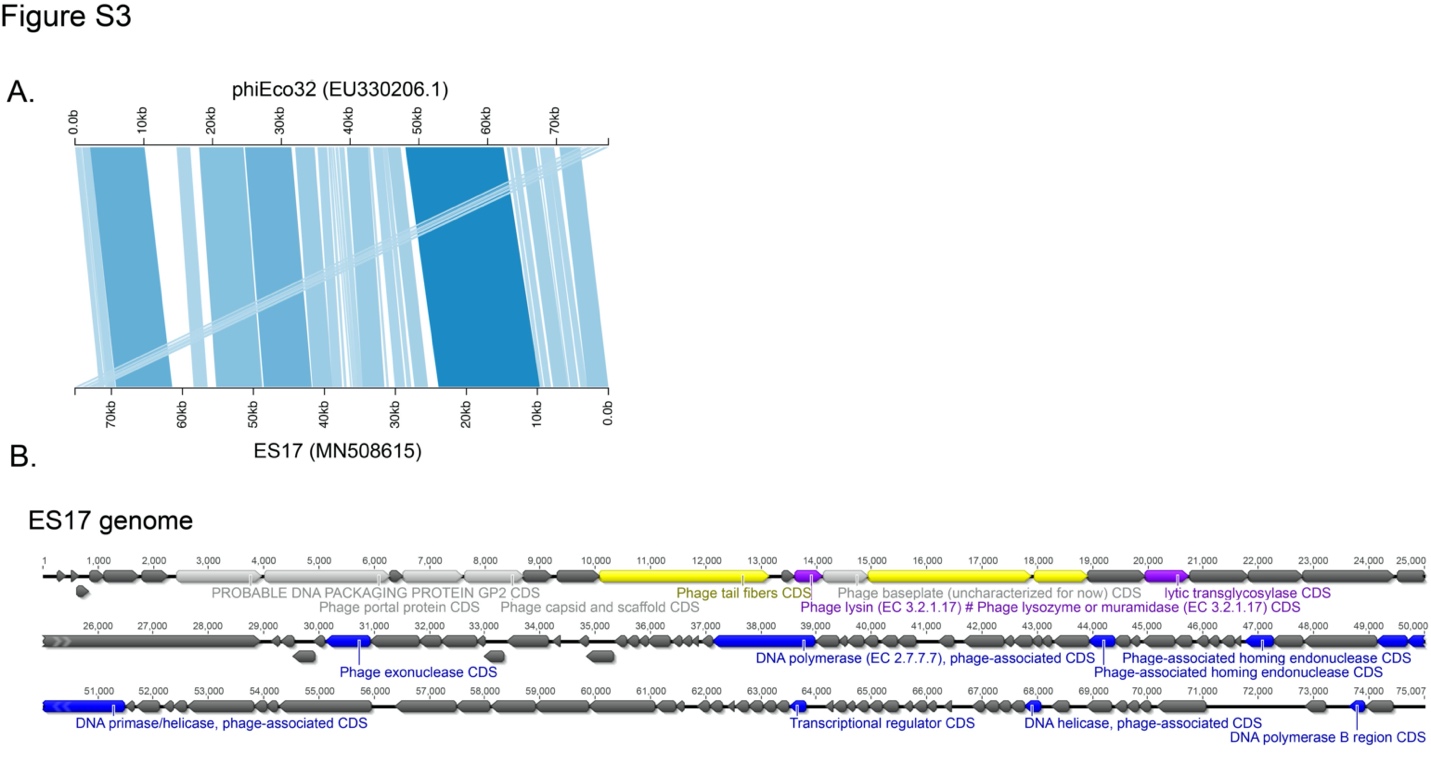


**Figure S3** (A) Graphical representation of BLAST genomic comparison analysis of phage ES17 (bottom axis; MN508615) and phage phiECo32 (top axis; EU330206.1) using Kablammo web-based software (1). Trapezoids drawn between the axis indicate individual BLAST alignments between the two sequences. The stronger alignments are shaded darker. (B) ES17 genome organization accession # MN508615.
