# Supplementary material for: Targeting of Mammalian Glycans Enhances Phage Predation in the Gastrointestinal Tract
Source: mBio. 2021 Feb 9;12(1):e03474-20. doi: 10.1128/mBio.03474-20 (PMC7885116; doi:10.1128/mBio.03474-20)
Supplement: FIG S4 [file mBio.03474-20-sf004.docx]

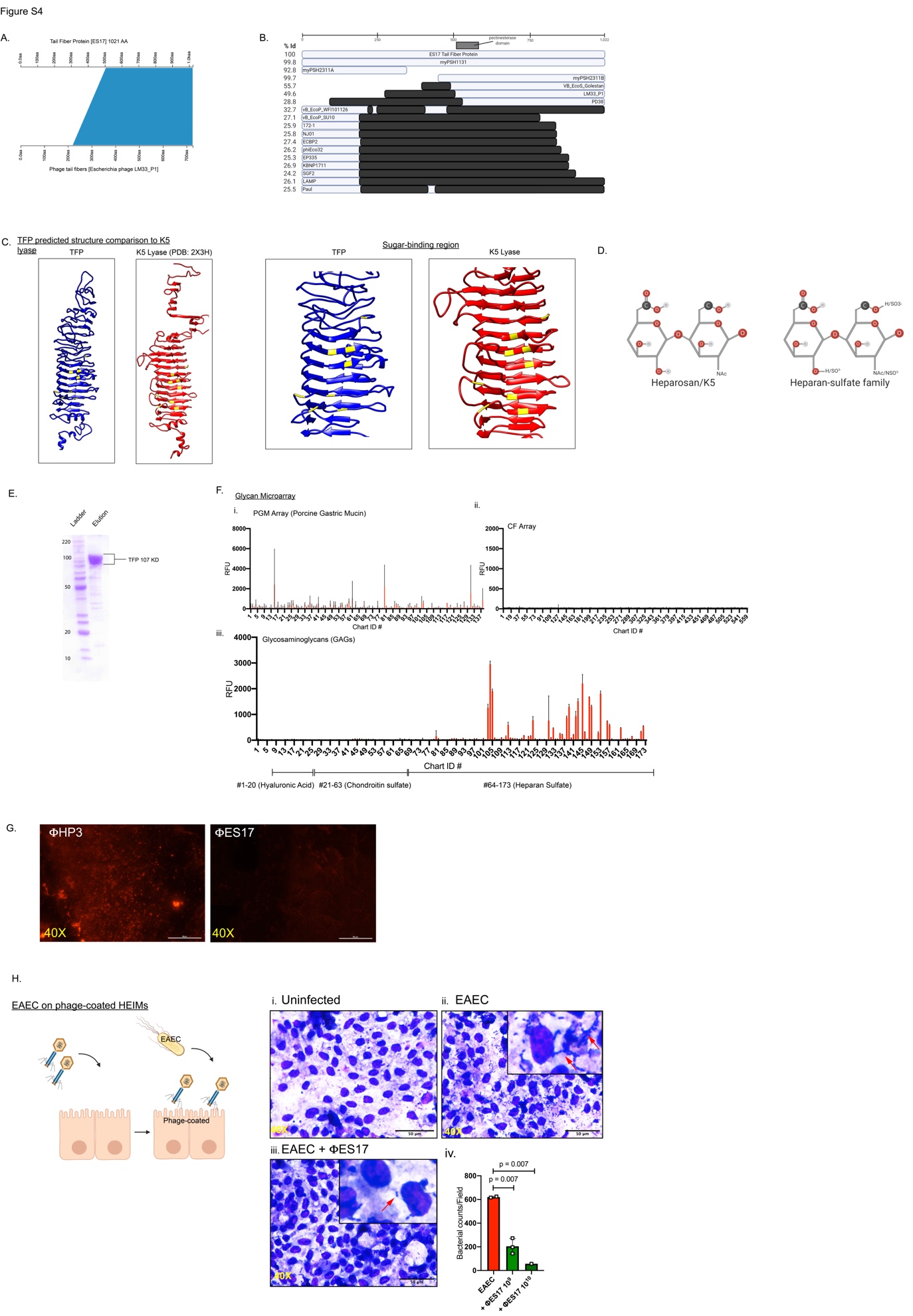


**Figure S4** (A) BLAST comparison analysis of ES17-TFP (top axis) and phage LM33_P1 tail fiber (bottom axis, ref|YP_009324518.1|).Trapezoids drawn between the axis indicate individual BLAST alignments between the two sequences. The stronger alignments are shaded darker. (B) ES17 TFP (Tail Fiber Protein; 3066 bp) pectinesterase domain blast analysis of showing similarity of domain to other phages. (C) Structures show the predicted structure of TFP tail fiber protein (blue), the structure of K5 lyase (PDB:2X3H; red). Identical residues between K5 lyase and ES17 tail fiber protein are yellow. Magnified sugar-binding domains shown in boxes. (D) Structures of heparosan or K5 capsule and heparan-sulfate derivative. (E) Protein SDS-PAGE gel showing purified ES-TFP pooled elutions and Benchmark Protein Ladder (lane 1). Pooled elutions from 2 Liter purification of TFP (lane 2). Approximate size of TFP 107 KD. (Fi) Glycan microarray analysis of 138 glycans from purified porcine gastric mucin in relative fluorescent units (RFUs) using purified ES17-TFP as the GBP (glycan binding protein).(Fii) Glycan array analysis of 560 defined glycans from the Consortium for Functional Glycomic using TFP as GBP. (Fiii) Glycan array analysis of 170 charged glycosaminoglycans (GAG), including oligomers from hyaluronic acid, chondroitin sulfates and heparan sulfate using TFP as GBP. (G) Immunofluorescent staining of ES17 and HP3 phages fixed on a slide using antibodies generated against HP3 (red). 40X magnification. (H). HIEMs (i) uninfected or (ii) Infected with EAEC 042 (iii) or pretreated with phage ES17 prior to EAEC. Images at 60X. Scale bars at 50 μm. (iv) EAEC attached to HIEMs per field view. Mean, ±SD shown. Squares indicate individual biological replicates from independent cultures. Figure created with BioRender.com.
