# Supplementary material for: Targeting of Mammalian Glycans Enhances Phage Predation in the Gastrointestinal Tract
Source: mBio. 2021 Feb 9;12(1):e03474-20. doi: 10.1128/mBio.03474-20 (PMC7885116; doi:10.1128/mBio.03474-20)
Supplement: TEXT S1 [file mBio.03474-20-s0001.docx]

**Supplemental Methods**

Antibiotic treated CM

For antibiotic treated cecal medium, CM was incubated with chloramphenicol (10 ug/mL, EMD Millipore) or ampicillin sodium salt (100 ug/mL) or ciprofloxacin (10 ug/mL) throughout the 4.5 hour infection using ExPEC JJ1901 (Figure S2A) or JJ2528 (Figure S2B) or commensal *E. coli* ECN (Figure S2C).

NAC Mouse Model

Mice (BALB/c) were housed individually and gavaged daily with NAC (N-acetyl cysteine) at a concentration of 40 mg/mouse/day throughout the experiment as indicated (14 days). This was a tolerable concentration and shown to be effective in the mouse small intestine (2). Mice were infected as described in the primary methods, then euthanized on day 6 and plated to determine bacterial concentration in the intestinal contents.

Phage Sequencing and Annotation

Phages were sequenced previously(3, 4). In brief, sequencing was performed on CsCl purified phages at the Center for Metagenomics and Microbiome Research (CMMR) at Baylor College of Medicine. Purified DNA samples were constructed into Illumina paired-end libraries. The libraries (avg. final size of 660 bp including adapter and barcode sequences) and were pooled in equimolar amounts to a final conc. 10 nM and templates prepared for sequencing on the Illumina MiSeq. After sequencing, the.bcl files were processed through Illumina’s analysis software (CASAVA), which demultiplexes pooled samples and generates sequence reads and base-call confidence values (qualities) (avg. raw yield/sample was 802 Mbp).

Assembly and Analysis

The adapter sequences were removed, and the sequence was then assembled using SPAdes v3.5.0 (5) on careful mode, retaining only contigs longer than 1,000 bp and with an avg. coverage of 1000x or greater. This generated 1–2 contigs per sample, with an average of 74% of the original reads mapping with 100% identity to the final contigs. Genomes were analyzed using both PATRIC’s comprehensive genome analysis service (6) and EDGE Bioinformatic software (7). Gene calling and genome annotation was performed using PROKKA (version 1.13) (8), RAST(9), (5), GLIMMER3 (version 3.02) (11) and GeneMarkS (version 4.28) (10). Annotated genome accession MN508615.

Genome Comparison

Genome comparison was performed using a BLAST analysis of phage ES17 (MN508615) and phiECO32 (EU330206.1) and visualized using Kablammo software (1).

Kuravirus Classification

Genus assignment for ES17 was based on sequence similarity, genomic arrangement, and morphology compared to multiple characterized Kuraviruses. ES17 is more than 95% identical (76 to 86% coverage) to: myPSH1311, myPSH2311, phiEco32, vB_EcoP_WFI101126, 172-1, O18-011, Paul, PGN6866, vB_EcoP_EcoN5, vB_EcoP_SU10, SGF2, NJ01, and LAMP.

ES17-TFP Construct Generation

The construct used to purify tail fiber protein (TFP) was prepared by Genewiz (South Plainfield, NJ) using established protocols. The construct sequence for TFP was synthesized from the phage ES17 sequence (MN508615.1) nucleotides 58,576-61,641, ttaacgcagggcacgtctactattagcgaagcgcatagcctctgcctgagtagtgaactccgctacgaagtaatcagcgaggcgtaggactactccgcctgtaccggctgtacttaggcgcaggttcactgaaccggaagcactagcctgagctacaccaaaggtactcacccactggccccccttagtaaatacttcacctacattgatatctccaccgatgcggaagtttgtaggggttccctgtaccagttcagtgctaatgccccatacataccagttaccggaagttatctgaaatgcagcaccagtaagctcatactcagcagcaccagggatttcgaagtacatgcagctctgagataactggccttgtggctgcaaactcccagtgctgcttgccgtaccactaaattcagcagtacctccatcgaagggtttaactaccagcttattaccaggtacacgatagatgtatccttccggcaggtatccacgcatactcatattaggtgtccagctttgcccaatcttgcctgtctgagccgctacagaacgtaccattacatctttgccggagctaccattcaggtacaattgatcagccacaaaactgctgttggcatctacagagatatccatgtggccatctgaattatctactcggcagccataagtaacaaggttggagttctgaagctctacgttattaaggtatgtgtactctgcaaagaagatactggtatcaataagtttaaggacacgaggcttctgagatacaccatctacattaatagctggagtagtagcgatagcttccatccatacattggatacaccacatggggcagtagggcagtcaccatccttgttcttgaggtaaataccaccaccgccagaggcttccataatactatctttaatccaccagccgcctacaccacctccgaatgtaccaggcgcttctcctgttgtagcgttcaagtaaacacagtaagtgctaataccatcaaagtgcatgttctgaagggtgtcagagcctaagtgcatctcagaagcagactgtgcccagtaaccccagtcacaaccttgcacgccaatgcctttccatgtattacctatattcccggaaggtttctgaatggccttgttaatgttatggatgtatacgtcactaaagttgtgacgaccagcatattggtcggtcgggtcaaaactaatacctacagccccggttgtcccagaaccatctatagcaaaaccaccactatggcggaattgccaatgaggcgtagatgccttaacctgaatagtgtctgtaaagtttgacggagctttcagtaccgtggctccaccaacaccctttaactgtgtactaccaatagtaatcgcttcactgtattcaccgggggaaacaattaagtcgttttttggtaacgatgcatttaccgcagcttgtaaggtagggaaatctcctgaatgcttaatgtcgttgttacggacaagcaaacttctctcaccactgccaatgtttttagccccatctttactggaaagactctcaacagaattaccattttcaaaggtaataagggatgcaccttcacctgcattactacttttcagccttgccagcaaatctgcttcacttgtatgcaactgtctcaaagcaaccgcgtcattatcatctacagcatcaggaattactggagattgtgtgaaagtaaccttgttggtcatcgaggtgatatcagagttatcaccactcttagctgcaactagagctagtcgagcattctctgatgaagtagctcctgtaccacctttggtaataggtacaattgattcagtggaaaccaaacctaaaccaagtttagtacgtgcagcacttgcagtagttgcccctgtgccaccctgagagatagtcacagggcgagcggaagacagctcttctcgtactgtaaatactcgggaaccaactcctccagtccagcttacagagaaaactgagtagtagttattattgtttgtccctggcaccactcgtacctcacctcctcctgtaactacgccaatgacttcaagatataacccataattggcaggataatttatctcagcaggggcatttgtaatattcgacattaatactgaatagttacctccattgacaaaattaaatgtttgccagtccatagccttcactttggttaaggtagggctaccaatcccatgctcagcgagtccaatatttgtaatagctgcacttgtgtcggttatttcggataggttattagttacactaaaagctcccacttgagcagctgtgtagtctccagtagcaggaactacagcacccttacgtccattgaatgtggagacagaatcaaaaaccactgaacctgtttgttcagcgccgccattgattgaaacagatcgaacagatggaatcaagttggcaatatctgacatgtcagctcggtataaaccaccatcatctttatctacatagaaaccatagtcatcaacaacaggggtaccaacaggaacgcttccaatatcaccgattgttccgtcagtaccatcacgccccggtttaccttctggaataccaaaagcaaagacacctgttgcattatcataagaccccgtagctaggcttcccggaggaagaagcgttgtttgaactgtcatgttagagataatgtcaagttgtgctagaacgttttgagttgcctcttttgaaagctcagttgctgcattggtgttgtcaattgcagttcctgtggattgaatcaaacctaagagcacttgttcttgtgactcaaagtcactcttgagttgtttaatgtcttcttccagtagatgaccttgagcatacagacgttctacttcagccaaaatctcctctactgaggagtatttagtttgggacaacaatgcccaatatttagagtctgctgcatattcagctgctgatttataagcaccaatagttccctcagtagctccaaattgtcctacattgttaacattcttaggtgcttggttgttataaacgatcat.

This sequence was ligated onto pET-21b (+) (ampicillin) vector using restriction enzymes 5’ *BamHI* and 3’ *Xhol* by recombination and delivered as a mini-scale DNA sample.

Protein Purification

BL21 (DE3) cells were used for expression of His_6_-TFP under the control of a T7 promoter expression. Cells were induced with Isopropyl ß-D-1-thiogalactopyranoside

(IPTG) 1mM for 2 hrs at 37°C. Then the culture was pelleted at 7000 RPM for 20 min and resuspended in Binding buffer (50mM NaH_2_PO_4_, pH 8.0 0.5 M NaCl). Cells were lysed using a French Press. Following lysing and centrifugation (15,000 RPM for 20 min.) the lysate was added to a Ni-NTA column for binding, washing 4 times in buffer (Binding buffer with 20mM imidazole) and elution (binding buffer with 250 mM imidazole). Buffer exchange was performed in PBS. An SDS-PAGE gel was run to verify the size of the protein along with a ladder (Benchmark Protein Ladder).

HIEM Generation

Human Intestinal Enteroid (HIEs) were cultured from 3D enteroid cultures derived from human colonic biopsy tissue using methods developed by Sato and Cleavers and 2-D enteroid monolayers (HEIMs) developed using methods of Van Dussen et al. (12–15). Briefly, HIEs were derived from human biopsy samples. The colonic crypts were isolated, embedded in Matrigel (Corning) or collagen (Sigma) and incubated with complete growth media with growth factors (CMGF+) for differentiation into 3D enteroid structures. Next, chambered slides (Greinier Bio-One) were coated with Matrigel or collagen, dissociated, about 50 spheroids, (3D enteroids cultured CMGF^+^ for 7 days) were added per well. HIEs were dissociated using 0.05% trypsin/0.5mM EDTA and a gravity flow strainer. Cells were allowed to grow and adhere 1-2 days with CMGF^-^. Then the media was removed and replaced with Differentiation media. Typically for experiment, HEIMs were used 4-7 days later when fully confluent ( >90%) and differentiated. For our experiments this was day 5. The components of media the CMGF+, CMGF- and Differentiation media are described previously (16).

EAEC infection and staining

The intestinal pathogen EAEC 042 (serotype 44:H18), was originally isolated from a child in Peru, and extensively characterized (17). For infection of HIEMs, the EAEC strain 042 was grown overnight and then sub-cultured (1:100) for 2 hrs at 37°C. One microliter of the log-phase bacterial culture was added to 100 microliters of the cell culture media and added to chambered slides either with phage-coated HIEMs (as described above) or untreated. Cultures were incubated for 3.5 hrs at 37°C in the presence of 5% CO_2_ in a humidified incubator. Following growth, bacteria were removed, by gently washing the cells in PBS then the remaining cells were fixed (Hema 3 fixative) and stained using a Geimsa-Wright stain (Fisher scientific). The infection and staining technique have been previously described (18).

Glycan Microarray

Glycan microarray analysis was performed by the Emory Comprehensive Glycomics Core using the methods described below.

An oligosaccharide array composed of 560 defined glycans from the Consortium for Functional Glycomics were obtained from MicroArray Core Facility of the Scripps Research Institute (La Jolla, CA) (lists can be found at <http://www.functionalglycomics.org/static/consortium/resources/resourcecored2.shtml#Glycolipid>) as described(19). The glycans were printed on Nexterion 3-D hydrogel coating (H) slides using a contact printer in replicates of six. A second microarray consisting of 138 glycans from purified porcine gastric mucin was also included. And a third microarray consisted of 170 charged glycosaminoglycans (GAG), including oligomers from hyaluronic acid, chondroitin sulfates and heparan, obtained from the Emory Comprehensive Glycomics Core (Atlanta, GA). Glycans were conjugated with the bifunctional linker (AEAB) and printed on Nexterion 3-D hydrogel coated (H) slides using a contact printer Aushon 2470 (Quanterix, Billerica, MA) in replicates of four. His-tagged TFP (up to 40 ug/mL) was diluted in PBS buffer with 1% BSA and 0.05% Tween 20 and incubated on the microarray slides for 1 hr at RT. Slides were washed with PBS buffer with 0.05% Tween 20 and PBS buffer. Alexa Flour 647 anti-His-tag mouse mAb (MBL International, Woburn, MA) was diluted to 5 µg/ml and incubated on the slides for 1 hr. After washing and drying, the slides were scanned with an InnoScan 1100 AL scanner and the data were processed using Mapix 8.2.5 software (Innopsys, Chicago, IL). Mean relative fluorescence units (RFU) from replicate spots were averaged after subtracting local median background, and the standard deviation (STDEV) were calculated and plotted as error bars in the histogram plot of the glycan array.

Bioinformatics modeling

The phage genome was annotated using the RASTtk (20). The Enzyme Function Initiative-Enzyme Similarity Tool (EFI-EST) was used to visualize BLAST results of the tail fiber protein (TFP) (21). The network visualization showed that TFP was related to other tail fiber proteins and proteins that belong to the pectin lyase fold/virulence factor family of proteins (IPR011050). HHpred was used to identify other proteins with similar structures (22). The highest scoring template was a Tailspike protein from *E. coli* bacteriophage HK620 (PDB: 2VJJ; E-value = 9.4E-23). The other top five hits from Enterobacteria or *Escherichia* phages were a tailspike protein from Enterobacteria phage SF6 (PDB: 2VBK; E-value=1.9E-14), a putative endo-glycosidase tailspike protein (PDB: 6NW9; E-value=3.1E-14), a colanidase tailspike protein from Enterobacteria phage phi92 (PDB:6E0V; E-value=1.2E-13), a sugar-binding tailspike from *Salmonella* phage Det7 (PDB:6F7D; E-value=2.1E-12), and a K5 lyase (E.C.4.2.2.7) tailspike protein from Enterobacteria phage K5 (PDB: 2X3H; E-value=4E-12). All of these tailspike proteins bind and degrade polysaccharides and are classified as members of the pectin lyase fold/virulence factor family (IPR011050)(23–27).The K5 lyase has well-known heparanase activity due to the similarities between K5 and heparan-sulfate (28). All six were used to produce a predicted structure of the ES17 tail fiber protein in Modeler (29). Structures were aligned using UCSF Chimera. Chimera’s matchmaker function was used to structurally align the predicted tail fiber 2 protein with the HK620 tailspike and the K5 lyase. Solvent exposed identical residues between ES17 tail fiber protein 2 and K5 lyase were determined using Chimera’s match-align to determine what residues were both identical and within 5 angstroms of each other in the structural alignment.

16S rRNA Gene Sequencing

The Center for Metagenomics and Microbiome Research (CMMR) at Baylor College of Medicine performed 16S rRNA sequencing and analysis.

The 16S rRNA gene sequencing methods were adapted from the methods developed for the Earth Microbiome Project (30) and NIH-Human Microbiome Project (30, 31). Briefly, bacterial genomic DNA was extracted using the Qiagen MagAttract PowerSoil DNA Kit (formerly sold by MO BIO as PowerMag Soil DNA Isolation Kit). The 16S rDNA V4 region was amplified by PCR using primers 515F and 806R containing Illumina adapters and a single-index barcodes (30). The amplicons were visualized with gel electrophoresis and purified to remove primer-dimers and non-specific amplicons. The samples were quantified using the Qubit® fluorometer and pooled at a DNA mass of 100 ng per sample. The amplicon pool was sequenced on the Illumina MiSeq platform using the reagent kit v2 (2 × 250 bp) and the paired-end protocol yielding paired-end reads that overlaped almost completely.

The raw data files in Binary Base Call (BCL) format created by the MiSeq run were first converted into the FASTQ format and demultiplexed based on the single-index barcodes using the Illumina BCL2FASTQ software. The demultiplexed FASTQ read pairs were then merged using USEARCH v7.0.1090 (32) ‘fastq_mergepairs’ function, requiring read pairs to overlap by at least 50 bp, a merged length of at least 252 bp, a truncation quality above 5, and zero differences in the overlapping region. The merged files were then filtered further, using the USEARCH70 ‘fastq_filter’ program, and only allowing for a maximum expected error of 0.05. Merged reads were then combined into a single FASTQ file, which was filtered for PhiX using Bowtie2 v.2.3.4.3 (33) and the ‘very-sensitive’ parameter setting. After removing PhiX reads, the FASTQ file was transformed into a FASTA format file. The FASTA file was run through USEARCH70 ‘derep_fulllength’ program and the reads were sorted by size using usearch70 ‘sortbysize’ program. The reads were clustered into operational taxonomic units (OTUs) at a similarity cutoff value of 97% using the UPARSE algorithm (34). This was accomplished in an iterative stepwise manner in increments of 0.4% using USEARCH70 ‘cluster_otus’ function. The output from the previous increment was fed into the next iteration, until a maximum of 3.2% was reached. All the intermediate files of this step were filtered for any chimeras and after the last run through the above loop, the final output was run through USEARCH70 ‘uchime_ref’ program against the GOLD database (35, 36), using only the plus strand and allowing for no chimeras, in order to create a clustered OTU file with no chimeras. The OTU file was mapped against an optimized version of the latest current SILVA Database (37) containing only sequences from the V4 region of the 16S rRNA gene to determine taxonomies. This step was performed using USEARCH70 ‘usearch_global’ function, specifying the identity threshold to 96.8%. Abundances were recovered by mapping the demultiplexed reads to the OUT file and all files created in the loop were then run through a program developed in-house that resolved the iterative UPARSE steps, creating an OTU table in BIOM format and removing the chimera and singleton reads. The BIOM file was summarized, recording the number of reads per sample, and merged with a file that was generated for the overall read statistics, to produce a final summary file with read statistics and taxonomy information. ATIMA (Agile Toolkit for Incisive Microbial Analyses) was used to generate beta diversity graphs. The Atima software is a web application combining publicly available R packages with purpose-written code to import sample data and identify trends in taxa abundance, alpha-diversity, and beta-diversity as they relate to sample metadata. This software explores correlations between taxonomic or functional profiles and sample metadata via highly-customizable plots and corresponding statistical analyses. Alpha diversity values (Shannon Diversity) were calculated based on the number and proportions of unique taxa.

**Supplemental References**

1. Wintersinger JA, Wasmuth JD. 2015. Kablammo: An interactive, web-based BLAST results visualizer. Bioinformatics https://doi.org/10.1093/bioinformatics/btu808.

2. De Lisle RC, Roach E, Jansson K. 2007. *Effects of laxative and N-acetylcysteine on mucus accumulation, bacterial load, transit, and inflammation in the cystic fibrosis mouse small intestine*. Am J Physiol 3:577-584.

3. Gibson SB, Green SI, Liu CG, Salazar KC, Clark JR, Terwilliger AL, Kaplan HB, Maresso AW, Trautner BW, Ramig RF. 2019. *Constructing and characterizing bacteriophage libraries for phage therapy of human infections*. Front Microbiol 10:2537.

4. Green SI, Kaelber JT, Ma L, Trautner BW, Ramig RF, Maresso AW. 2017. *Bacteriophages from ExPEC reservoirs kill pandemic multidrug-resistant strains of clonal group ST131 in animal models of bacteremia*. Sci Rep 7:46151.

5. Bankevich A, Nurk S, Antipov D, Gurevich AA, Dvorkin M, Kulikov AS, Lesin VM, Nikolenko SI, Pham S, Prjibelski AD, Pyshkin A V., Sirotkin A V., Vyahhi N, Tesler G, Alekseyev MA, Pevzner PA. 2012. *SPAdes: A new genome assembly algorithm and its applications to single-cell sequencing*. J Comput Biol 5:455-477.

6. Wattam AR, Davis JJ, Assaf R, Boisvert S, Brettin T, Bun C, Conrad N, Dietrich EM, Disz T, Gabbard JL, Gerdes S, Henry CS, Kenyon RW, Machi D, Mao C, Nordberg EK, Olsen GJ, Murphy-Olson DE, Olson R, Overbeek R, Parrello B, Pusch GD, Shukla M, Vonstein V, Warren A, Xia F, Yoo H, Stevens RL. 2017. *Improvements to PATRIC, the all-bacterial bioinformatics database and analysis resource center*. Nucleic Acids Res https://doi.org/10.1093/nar/gkw1017.

7. Li PE, Lo CC, Anderson JJ, Davenport KW, Bishop-Lilly KA, Xu Y, Ahmed S, Feng S, Mokashi VP, Chain PSG. 2017. *Enabling the democratization of the genomics revolution with a fully integrated web-based bioinformatics platform*. Nucleic Acids Res 1:67-80.

8. Lo CC, Chain PSG. 2014. *Rapid evaluation and quality control of next generation sequencing data with FaQCs*. BMC Bioinformatics 15:366.

9. Zerbino DR, Birney E. 2008. *Velvet: algorithms for de novo short read assembly using de bruijn graphs*. Genome Res 5:821-829.

10. Peng Y, Leung HCM, Yiu SM, Chin FYL. 2012. IDBA-UD: *A de novo assembler for single-cell and metagenomic sequencing data with highly uneven depth*. Bioinformatics 28:1420-1428.

11. Peng Y, Leung HCM, Yiu SM, Chin FYL. 2010. IDBA – A Practical Iterative de Bruijn Graph De Novo Assembler. In: Berger B. (eds) *Research in Computational Molecular Biology*. RECOMB 2010. Lecture Notes in Computer Science, vol 6044. Springer, Berlin, Heidelberg. https://doi.org/10.1007/978-3-642-12683-3_28

12. Sato T, Stange DE, Ferrante M, Vries RGJ, Van Es JH, Van Den Brink S, Van Houdt WJ, Pronk A, Van Gorp J, Siersema PD, Clevers H. 2011. *Long-term expansion of epithelial organoids from human colon, adenoma, adenocarcinoma, and Barrett’s epithelium*. Gastroenterology 5:1762-1772.

13. Sato T, Vries RG, Snippert HJ, Van De Wetering M, Barker N, Stange DE, Van Es JH, Abo A, Kujala P, Peters PJ, Clevers H. 2009. *Single Lgr5 stem cells build crypt-villus structures in vitro without a mesenchymal niche*. Nature 14:262-265.

14. VanDussen KL, Marinshaw JM, Shaikh N, Miyoshi H, Moon C, Tarr PI, Ciorba MA, Stappenbeck TS. 2015. *Development of an enhanced human gastrointestinal epithelial culture system to facilitate patient-based assays*. Gut 6:911-920.

15. Saxena K, Blutt SE, Ettayebi K, Zeng XL, Broughman JR, Crawford SE, Karandikar UC, Sastri NP, Conner ME, Opekun AR, Graham DY, Qureshi W, Sherman V, Foulke-Abel J, In J, Kovbasnjuk O, Zachos NC, Donowitz M, Estes MK. 2016. *Human intestinal enteroids: a new model to study human rotavirus infection, host restriction, and pathophysiology*. J Virol 90:43–56.

16. Poole NM, Rajan A, Maresso AW. 2018. *Human intestinal enteroids for the study of bacterial adherence, invasion, and translocation*. Curr Protoc Microbiol 50:e55.

17. Nataro JP, Baldini MM, Kaper JB, Black RE, Bravo N, Levine MM. 1985. *Detection of an adherence factor of enteropathogenic escherichia coli with a dna probe*. J Infect Dis 3:560-565.

18. Rajan A, Vela L, Zeng XL, Yu X, Shroyer N, Blutt SE, Poole NM, Carlin LG, Nataro JP, Estes MK, Okhuysen PC, Maresso AW. 2018. *Novel segment- and host-specific patterns of enteroaggregative escherichia coli adherence to human intestinal enteroids*. mBio 1:e02419-17.

19. Heimburg-Molinaro J, Song X, Smith DF, Cummings RD. 2011. *Preparation and analysis of glycan microarrays*. Curr Protoc Protein Sci https://doi.org/10.1002/0471140864.ps1210s64.

20. Brettin T, Davis JJ, Disz T, Edwards RA, Gerdes S, Olsen GJ, Olson R, Overbeek R, Parrello B, Pusch GD, Shukla M, Thomason JA, Stevens R, Vonstein V, Wattam AR, Xia F. 2015. *RASTtk: a modular and extensible implementation of the RAST algorithm for building custom annotation pipelines and annotating batches of genomes*. Sci Rep 5:8365.

21. Gerlt JA, Bouvier JT, Davidson DB, Imker HJ, Sadkhin B, Slater DR, Whalen KL. 2015. *Enzyme Function Initiative-Enzyme Similarity Tool (EFI-EST): A web tool for generating protein sequence similarity networks*. Biochim Biophys Acta 1854:1019–1037.

22. Hildebrand A, Remmert M, Biegert A, Soding J. 2009. *Fast and accurate automatic structure prediction with HHpred*. Proteins 77 Suppl 9:128–132.

23. Freiberg A, Morona R, den Bosch L, Jung C, Behlke J, Carlin N, Seckler R, Baxa U. 2003. *The tailspike protein of Shigella phage Sf6. A structural homolog of Salmonella phage P22 tailspike protein without sequence similarity in the beta-helix domain*. J Biol Chem 278:1542–1548.

24. Barbirz S, Muller JJ, Uetrecht C, Clark AJ, Heinemann U, Seckler R. 2008. *Crystal structure of Escherichia coli phage HK620 tailspike: podoviral tailspike endoglycosidase modules are evolutionarily related*. Mol Microbiol 69:303–316.

25. Greenfield J, Shang X, Luo H, Zhou Y, Heselpoth RD, Nelson DC, Herzberg O. 2019. *Structure and tailspike glycosidase machinery of ORF212 from E. coli O157:H7 phage CBA120 (TSP3)*. Sci Rep 9:7349.

26. Schwarzer D, Buettner FF, Browning C, Nazarov S, Rabsch W, Bethe A, Oberbeck A, Bowman VD, Stummeyer K, Muhlenhoff M, Leiman PG, Gerardy-Schahn R. 2012. *A multivalent adsorption apparatus explains the broad host range of phage phi92: a comprehensive genomic and structural analysis*. J Virol 86:10384–10398.

27. Broeker NK, Roske Y, Valleriani A, Stephan MS, Andres D, Koetz J, Heinemann U, Barbirz S. 2019. *Time-resolved DNA release from an O-antigen-specific*. J Biol Chem 294:11751–11761.

28. O’Leary TR, Xu Y, Liu J. 2013. *Investigation of the substrate specificity of K5 lyase A from K5A bacteriophage*. Glycobiology 23:132–141.

29. Zimmermann L, Stephens A, Nam SZ, Rau D, Kubler J, Lozajic M, Gabler F, Soding J, Lupas AN, Alva V. 2018. *A Completely Reimplemented MPI Bioinformatics Toolkit with a New HHpred Server at its Core*. J Mol Biol 430:2237–2243.

30. Caporaso JG, Lauber CL, Walters WA, Berg-Lyons D, Huntley J, Fierer N, Owens SM, Betley J, Fraser L, Bauer M, Gormley N, Gilbert JA, Smith G, Knight R. 2012. *Ultra-high-throughput microbial community analysis on the Illumina HiSeq and MiSeq platforms*. ISME J 6:1621-1624.

31. Huttenhower C, Gevers D, Knight R, Abubucker S, Badger JH, Chinwalla AT, Creasy HH, Earl AM, Fitzgerald MG, Fulton RS, Giglio MG, Hallsworth-Pepin K, Lobos EA, Madupu R, Magrini V, Martin JC, Mitreva M, Muzny DM, Sodergren EJ, Versalovic J, Wollam AM, Worley KC, Wortman JR, Young SK, Zeng Q, Aagaard KM, Abolude OO, Allen-Vercoe E, Alm EJ, Alvarado L, Andersen GL, Anderson S, Appelbaum E, Arachchi HM, Armitage G, Arze CA, Ayvaz T, Baker CC, Begg L, Belachew T, Bhonagiri V, Bihan M, Blaser MJ, Bloom T, Bonazzi V, Paul Brooks J, Buck GA, Buhay CJ, Busam DA, Campbell JL, Canon SR, Cantarel BL, Chain PSG, Chen IMA, Chen L, Chhibba S, Chu K, Ciulla DM, Clemente JC, Clifton SW, Conlan S, Crabtree J, Cutting MA, Davidovics NJ, Davis CC, Desantis TZ, Deal C, Delehaunty KD, Dewhirst FE, Deych E, Ding Y, Dooling DJ, Dugan SP, Michael Dunne W, Scott Durkin A, Edgar RC, Erlich RL, Farmer CN, Farrell RM, Faust K, Feldgarden M, Felix VM, Fisher S, Fodor AA, Forney LJ, Foster L, Di Francesco V, Friedman J, Friedrich DC, Fronick CC, Fulton LL, Gao H, Garcia N, Giannoukos G, Giblin C, Giovanni MY, Goldberg JM, Goll J, Gonzalez A, Griggs A, Gujja S, Kinder Haake S, Haas BJ, Hamilton HA, Harris EL, Hepburn TA, Herter B, Hoffmann DE, Holder ME, Howarth C, Huang KH, Huse SM, Izard J, Jansson JK, Jiang H, Jordan C, Joshi V, Katancik JA, Keitel WA, Kelley ST, Kells C, King NB, Knights D, Kong HH, Koren O, Koren S, Kota KC, Kovar CL, Kyrpides NC, La Rosa PS, Lee SL, Lemon KP, Lennon N, Lewis CM, Lewis L, Ley RE, Li K, Liolios K, Liu B, Liu Y, Lo CC, Lozupone CA, Dwayne Lunsford R, Madden T, Mahurkar AA, Mannon PJ, Mardis ER, Markowitz VM, Mavromatis K, McCorrison JM, McDonald D, McEwen J, McGuire AL, McInnes P, Mehta T, Mihindukulasuriya KA, Miller JR, Minx PJ, Newsham I, Nusbaum C, Oglaughlin M, Orvis J, Pagani I, Palaniappan K, Patel SM, Pearson M, Peterson J, Podar M, Pohl C, Pollard KS, Pop M, Priest ME, Proctor LM, Qin X, Raes J, Ravel J, Reid JG, Rho M, Rhodes R, Riehle KP, Rivera MC, Rodriguez-Mueller B, Rogers YH, Ross MC, Russ C, Sanka RK, Sankar P, Fah Sathirapongsasuti J, Schloss JA, Schloss PD, Schmidt TM, Scholz M, Schriml L, Schubert AM, Segata N, Segre JA, Shannon WD, Sharp RR, Sharpton TJ, Shenoy N, Sheth NU, Simone GA, Singh I, Smillie CS, Sobel JD, Sommer DD, Spicer P, Sutton GG, Sykes SM, Tabbaa DG, Thiagarajan M, Tomlinson CM, Torralba M, Treangen TJ, Truty RM, Vishnivetskaya TA, Walker J, Wang L, Wang Z, Ward D V., Warren W, Watson MA, Wellington C, Wetterstrand KA, White JR, Wilczek-Boney K, Wu Y, Wylie KM, Wylie T, Yandava C, Ye L, Ye Y, Yooseph S, Youmans BP, Zhang L, Zhou Y, Zhu Y, Zoloth L, Zucker JD, Birren BW, Gibbs RA, Highlander SK, Methé BA, Nelson KE, Petrosino JF, Weinstock GM, Wilson RK, White O. 2012. *Structure, function and diversity of the healthy human microbiome*. Nature 486:207-214.

32. Edgar RC. 2010. Search and clustering orders of magnitude faster than BLAST. Bioinformatics 26:2460-2461.

33. Langmead B, Salzberg SL. 2012. *Fast gapped-read alignment with Bowtie 2*. Nat Methods 9:357-359.

34. Edgar RC. 2013. *UPARSE: Highly accurate OTU sequences from microbial amplicon reads*. Nat Methods 10:996-998.

35. Kyrpides NC. 1999. *Genomes Online Database (GOLD 1.0): A monitor of complete and ongoing genome projects world-wide*. Bioinformatics.15:773-774.

36. Mukherjee S, Stamatis D, Bertsch J, Ovchinnikova G, Katta HY, Mojica A, Chen IMA, Kyrpides NC, Reddy TBK. 2019. *Genomes OnLine database (GOLD) v.7: Updates and new features*. Nucleic Acids Res 46:649-659.

37. Quast C, Pruesse E, Yilmaz P, Gerken J, Schweer T, Yarza P, Peplies J, Glöckner FO. 2013. *The SILVA ribosomal RNA gene database project: Improved data processing and web-based tools*. Nucleic Acids Res 41:590-596.
