# Supplementary material for: Targeting of Mammalian Glycans Enhances Phage Predation in the Gastrointestinal Tract
Source: mBio. 2021 Feb 9;12(1):e03474-20. doi: 10.1128/mBio.03474-20 (PMC7885116; doi:10.1128/mBio.03474-20)
Supplement: FIG S5 [file mBio.03474-20-sf005.docx]

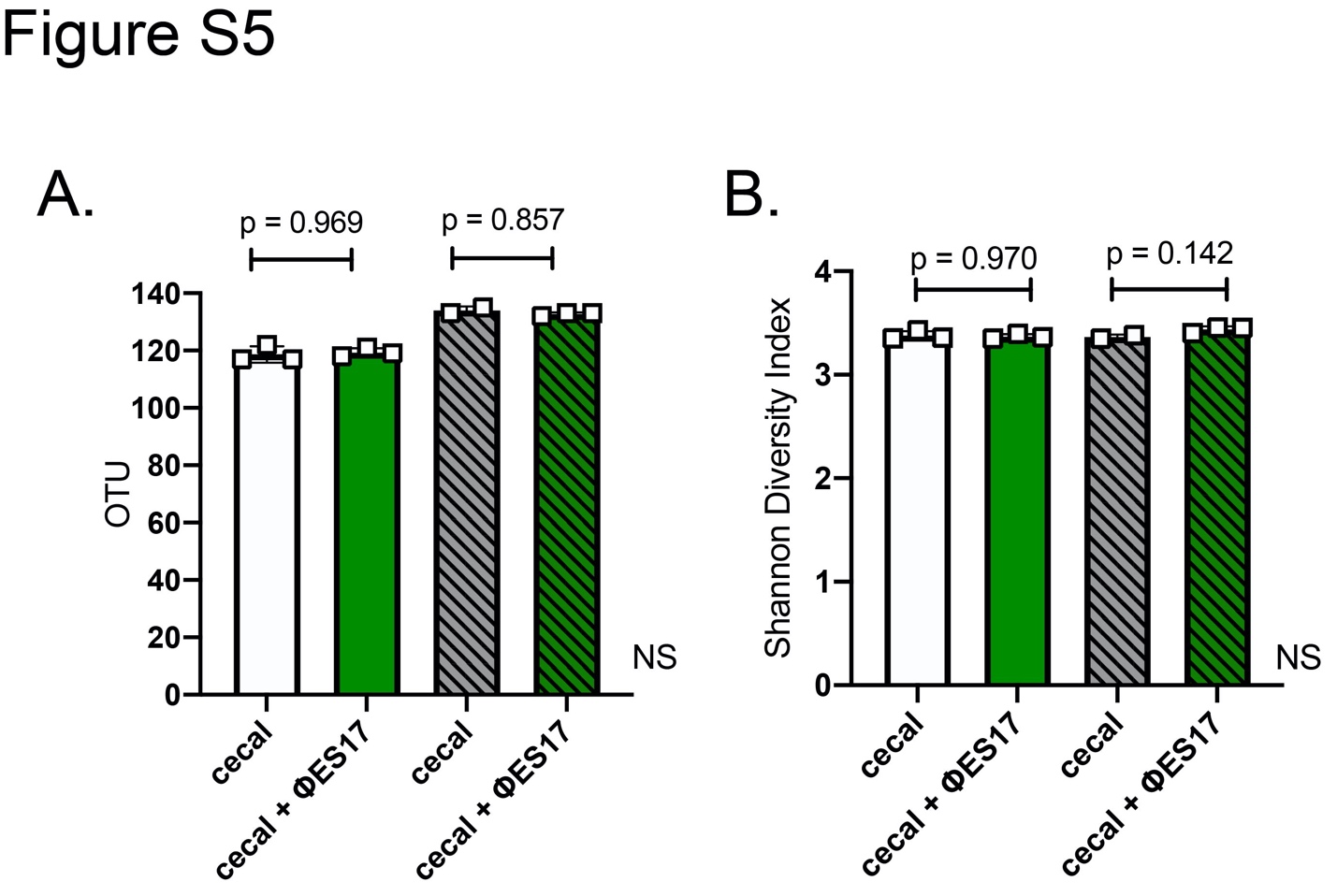


**Figure S5** (A) OTU from 16S rDNA analysis of cecal media after incubation with phage ES17. (B) Shannon diversity index values shown of cecal media after incubation with phage ES17. N=3. Groups: Cecal (white), Cecal + ΦES17 (green), anaerobic cecal (grey, striped), anaerobic cecal + ΦES17 (grey, striped + ΦES17 (green).

Mean, ±SD shown. Squares rep. indep. cultures. One-way ANOVA used for significance. NS = not significant.
